# Radiation-dose-dependent functional synergisms between ATM, ATR and DNA-PKcs in checkpoint control and resection in G_2_-phase

**DOI:** 10.1038/s41598-019-44771-6

**Published:** 2019-06-04

**Authors:** Emil Mladenov, Xiaoxiang Fan, Rositsa Dueva, Aashish Soni, George Iliakis

**Affiliations:** Institute of Medical Radiation Biology, University of Duisburg-Essen Medical School, 45122 Essen, Germany

**Keywords:** Double-strand DNA breaks, DNA damage checkpoints

## Abstract

Using data generated with cells exposed to ionizing-radiation (IR) in G_2_-phase of the cell cycle, we describe dose-dependent interactions between ATM, ATR and DNA-PKcs revealing unknown mechanistic underpinnings for two key facets of the DNA damage response: DSB end-resection and G_2_-checkpoint activation. At low IR-doses that induce low DSB-numbers in the genome, ATM and ATR regulate epistatically the G_2_-checkpoint, with ATR at the output-node, interfacing with the cell-cycle predominantly through Chk1. Strikingly, at low IR-doses, ATM and ATR epistatically regulate also resection, and inhibition of either activity fully suppresses resection. At high IR-doses that induce high DSB-numbers in the genome, the tight ATM/ATR coupling relaxes and independent outputs to G_2_-checkpoint and resection occur. Consequently, both kinases must be inhibited to fully suppress checkpoint activation and resection. DNA-PKcs integrates to the ATM/ATR module by regulating resection at all IR-doses, with defects in DNA-PKcs causing hyper-resection and G_2_-checkpoint hyper-activation. Notably, hyper-resection is absent from other c-NHEJ mutants. Thus, DNA-PKcs specifically regulates resection and adjusts the activation of the ATM/ATR module. We propose that selected DSBs are shepherd by DNA-PKcs from c-NHEJ to resection-dependent pathways for processing under the regulatory supervision of the ATM/ATR module.

## Introduction

The network of cellular responses to DNA damage (DDR)^1–3^ is built around three kinases of the phosphoinositide-3-kinase (PI3K)-related family of protein kinases (PIKKs): ataxia-telangiectasia mutated (ATM), ATM and RAD3-related (ATR) and DNA-dependent protein kinase, catalytic subunit (DNA-PKcs)^4^. ATM, ATR, and DNA-PKcs are large polypeptides with similar domain organizations and common structural features, including a C-terminally located kinase domain and N-terminally located HEAT-repeat domains that mediate protein-protein interactions^5^. They all show preference for phosphorylating serine or threonine residues followed by a glutamine (S/T-Q) and share as a result substrates and functions in DDR. DNA-PKcs^6–8^, ATM^9,10^ and ATR^11–13^ undergo autophosphorylation and there is evidence, particularly for DNA-PKcs^6^, that this event is important in their regulation^5^. Recruitment of ATM, ATR, and DNA-PKcs to DNA damage sites is regulated by protein co-factors that are specific to each kinase, but which share a related C-terminal motif thought to interact with their HEAT repeats. ATM is recruited by NBS1^14^, ATR by ATRIP^15^, and DNA-PKcs by Ku80^16,17^.

DNA-PKcs acts as a sensor for DSBs through recruitment by Ku that causes its activation^6^. Activated DNA-PKcs phosphorylates various substrates including itself and other c-NHEJ factors, and acts by facilitating the formation of short-range synaptic complexes^18^. It has a major role in promoting classical non-homologous end-joining (c-NHEJ), considered the main pathway for repairing DSBs throughout the cell cycle^19,20^, with a presumed, small contribution being made by homologous recombination repair (HRR) during S and G_2_-phase^21,22^.

ATM is the apical kinase thought to be regulating, through phosphorylation of hundreds of substrates^23^, the global cellular responses initiated by DSBs, including the coordination of DSB repair events and the activation of cell cycle checkpoints^24,25^. A relevant substrate of ATM is the Chk2 kinase, which is activated by ATM and phosphorylated on multiple sites including T68^26–29^, that is routinely used as evidence for ATM activation. However, mainly apoptosis is reduced in Chk2-deficient cells, while checkpoint signaling remains largely intact^30–33^.

ATM is recruited to DSBs by the Mre11-Rad50-Nbs1 (MRN) complex^34^ that stimulates its kinase activity^35,36^. ATM activation is further promoted by a C-terminal acetylation induced by DSB-associated chromatin changes exposing the H3K9me3 histone mark that recruits and activates, via shielding from dephosphorylation^37^, the TIP60/Kat9 acetyltransferase^38^. Notably, the kinase of TIP60/Kat9 acetyltransferase is c-Abl^37^, itself an ATM substrate^39,40^.

ATM rapidly initiates and sustains chromatin-based DDR signaling through phosphorylation of S139 of histone variant H2AX to generate γ-H2AX. γ-H2AX initiates a chromatin-based signaling cascade involving phosphorylation, ubiquitylation, and other post-translational modifications in proteins involved in DDR^41–44^. Ultimate goal of the ensuing events in the vicinity of the DSB, is the spreading along chromatin of DDR factor-assembly that amplifies the initial signal and promotes the recruitment of the scaffold protein 53BP1 to channel DSB repair toward c-NHEJ. The function of 53BP1 is antagonized by BRCA1, a protein favoring DSB processing by HRR, through mechanisms that are being elucidated^44^.

Assisted by Artemis, ATM is thought to regulate processing of a subset of DSBs via c-NHEJ^45^, a function it may partly exercise by facilitating DSB-end bridging^46^. ATM also promotes DSB repair by HRR. In this function, it is primarily involved in the regulation of DNA-end resection^33,47–50^, in part through phosphorylation of the key resection factor CtIP^51–53^. Despite these connections, however, ATM is not essential for HRR.

ATR is the apical DNA replication-stress-response kinase^54^ and unlike ATM and DNA-PKcs, it is essential in proliferating cells^55,56^. ATR is activated by a wide range of genotoxic stresses, owing to its recruitment by ATRIP to extended tracts of ssDNA coated with the ssDNA binding protein complex, replication protein A (RPA)^15^. Such RPA-coated ssDNA is generated by nucleolytic processing of various forms of damaged DNA^57^ or by helicase-polymerase uncoupling at stalled replication forks^58^. Notable and relevant here is the ATR activation induced at resected DSBs.

Full ATR activation also requires ssDNA/dsDNA junctions and activator proteins, such as TopBP1^59^, whose ATR activation domain contacts both ATRIP and the C-terminus of ATR^60^. TopBP1 also binds to the C-terminal tail of the RAD9 subunit of the RAD9-RAD1-HUS1 (9-1-1) complex^61,62^. Recently, a second ATR-activator protein, ETAA1, that is recruited to RPA-ssDNA via direct binding to RPA was identified and found to contain an ATR-activation domain similar to that of TopBP1^63–65^; thus, the full activation of ATR is likely to be complex and highly context dependent. An important function of ATR is to phosphorylate and activate the protein kinase Chk1^66–69^, which in S-phase cells inactivates the Cdc25A and in G_2_–phase the Cdc25C phosphatase that removes inhibitory modifications from cyclin dependent kinases (CDKs)^70,71^ and initiates cell cycle phase transitions.

Despite the functional differentiation among ATM, ATR and DNA-PKcs, an integrating characteristic that adds to their structural and functional similarities, is their connection with and activation at the DSB; and most importantly their involvement in DSB processing and the complex signaling emanating from it. Such single-lesion-dedication brings DNA-PKcs, ATM and ATR to specific locations in the nucleus at well-defined times, and generates opportunities for crosstalk that has only recently begun to be functionally characterized.

Indeed, several regulatory connections have been identified between DNA-PKcs and ATM. Expression of ATM is regulated by DNA-PKcs such that ATM protein levels are decreased in DNA-PKcs-deficient cells^72^. Also, DNA-PKcs regulates negatively ATM activity through phosphorylation at multiple sites^73^, and, consistently, loss of DNA-PKcs function leads to hyperactivation of ATM and amplification of the p53 response^74^. Vice-versa, DNA-PKcs is phosphorylated by ATM at residues T2609 and T2647 in response to DNA damage^75^ and this phosphorylation helps to overcome Ku/DNA-PKcs inhibition of resection *in vitro*^76^. Crosstalk has also been documented between DNA-PKcs and ATM during V(D)J recombination, where functions of DNA-PKcs, beyond Artemis activation, can be redundantly assumed by ATM^77^. Similarly, in cells undergoing immunoglobulin class switch recombination, regulatory functions of ATM can in its absence also be carried out by DNA-PKcs^78^.

Crosstalk between ATM and ATR has also been reported. Notably, ATM-dependent resection is known to facilitate ATR activation in the S- and G_2_ –phase of the cell cycle, suggesting a directional crosstalk between ATM and ATR in response to DSBs^33,49,50^. Such a sequence of events that leads to resection-dependent and mutually exclusive activation states for the two kinases has been termed the ATM to ATR switch and analyzed in biochemical studies^79^.

In addition, ATM-deficient cells enforce an ATR-dependent G_2_/M-checkpoint^80,81^ and show other functions that are dependent on DNA-PKcs, suggesting crosstalk between DNA-PKcs and ATR^81^. Also, activation of endogenous ATR and Chk1 in human cell-free extracts depends under certain conditions on DNA-PKcs that acts by phosphorylating RPA32 and TopBP1^82^. DNA-PKcs helps to minimize the adverse consequences of ATR-inhibition-associated replication stress^83^ and facilitates ATR-Chk1 signaling^84^. Finally, and in line with their recruitment to the DSB site, DNA-PKcs associates with ATR *in vivo* and is phosphorylated by ATR *in vitro*^85^.

The evidence outlined above for crosstalk and functional overlap between DNA-PKcs, ATM and ATR under pathological conditions, suggests a modular integration of the three kinases, which under homeostatic conditions will ensure sustained PIKKs-signaling even when DSB processing takes the DNA ends through states activating only one of these kinases. In the present paper we outline an in depth analysis of the modular integration of DNA-PKcs, ATM and ATR in two key aspects of DDR: The activation of the G_2_–checkpoint^86^ and the regulation of resection^87^. A unique characteristic of the study is the specific analysis of adaptations that we discovered in the crosstalk between DNA-PKcs, ATM and ATR as a function of the DSB load in the cellular genome. The importance of damage load in DDR is underscored by recent reports that ATR, by suppressing dormant origins, prevents exhaustion of nuclear RPA and avoids destabilizing DNA damage in replicating genomes^88^; and that 53BP1-mediated suppression of hyper-resection limits mutagenic pathways of DSB processing that fuel genome instability^89^.

## Results

### Unique pairing of ATM and ATR in a module regulating checkpoint in G_2_-phase cells exposed to low doses of IR

We focused our experiments on the activation of the DNA damage checkpoint and the induction of resection at DSBs in cells exposed to IR in the G_2_–phase of the cell cycle, where all DSB processing pathways are functional. In addition, we studied systematically these endpoints as a function of IR dose, i.e. DSB load, which enabled us to uncover mechanistic underpinnings of an important facet of DDR that has only recently begun to receive attention^88,89^. In the following sections we discuss separately experiments carried out in the low-dose range, defined here as doses between 0–4 Gy, and in the high-dose range, defined as doses between 5 and 20 Gy.

**Figure 1:**
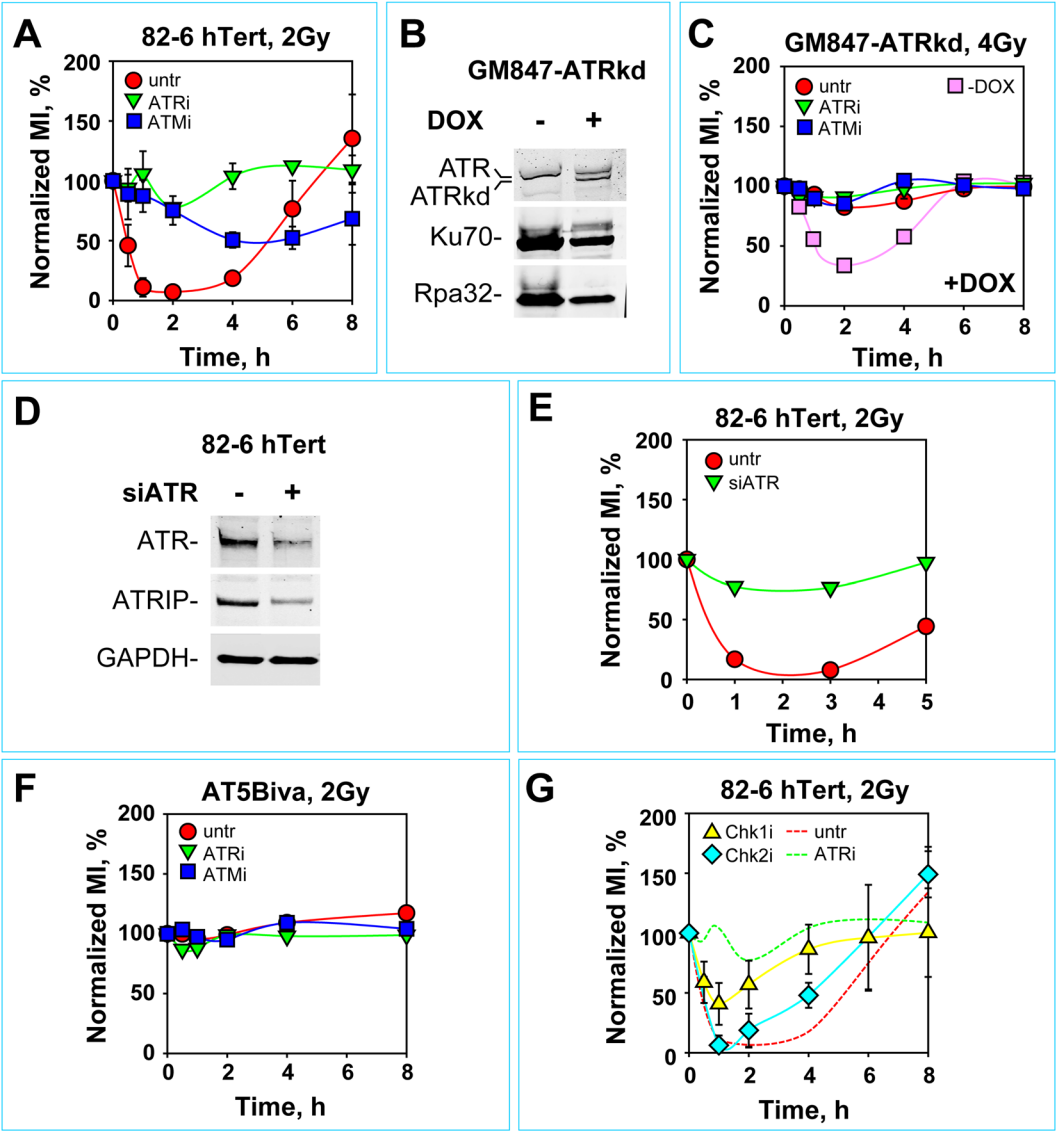
ATM and ATR are both essential for the manifestation of checkpoint in G_2_-cells exposed to low IR doses. (**A**) Normalized mitotic index (MI) calculated from bivariate PI, histone H3-pS10 flow cytometry in 82-6 hTert cells; untreated (untr) or treated with indicated PIKK inhibitors. The values of MI used for normalization in different experiments were: MI_untr_ = (2.75–3.05%), MI_ATRi_ = (3.76–5.62%), MI_ATMi_ = (3.19–6.15%). Mainly cells irradiated in G_2_ reach M-phase within the observation time. (**B**) Western blot analysis of GM847-ATRkd cells, showing ATRkd expression 48 h after incubation with 3 µg/ml doxycycline (DOX). Bands corresponding to ATR and ATRkd are indicated. The signals of Ku70 and Rpa32 serve as loading controls. (**C**) Normalized MI as function of time after exposure to 4 Gy of DOX-treated (+DOX) GM847-ATRkd cells. The response of cells analyzed in the absence of DOX (−DOX) are also shown for comparison. (**D**) Western blot analysis of ATR and ATRIP in 82–6 hTert cells, 48 h after mock-transfection or transfection with siRNA targeting ATR. GAPDH serves as a loading control. (**E**) Normalized MI as a function of time after exposure to 2 Gy of 82-6 hTert cells transfected with siATR 48 h prior to irradiation. Shown are results of one representative experiment. (**F**) Normalized MI as a function of time after exposure of AT5Biva cells to 2 Gy, in the presence of ATMi or ATRi. The values of MI used for normalization in different experiments were: MI_untr_ = (2.52–3.04%), MI_ATRi_ = (2.78–4.02), MI_ATMi_ = (2.44–3.18%). Other details as in (**A**). (**G**) Normalized MI as a function of time after exposure to 2 Gy of 82-6 hTert cells, treated with Chk1 or Chk2 inhibitors as indicated. Dotted lines show for comparison results obtained in the absence of any treatment (untr) or after treatment with ATRi. Unless otherwise stated, all data points show the mean and standard deviation (visible only when larger than the symbol) calculated from three independent experiments.

To study specifically the G_2_-checkpoint in the G_2_-fraction of an exponentially proliferating culture of irradiated cells, we adopted a frequently applied two-parameter flow cytometry method combining DNA and histone H3 phospho-Serine 10 (H3-pS10) detection to identify mitotic cells^90–92^ and quantitate the mitotic index (MI). Exposure of actively proliferating hTert immortalized normal human fibroblasts 82–6 (82–6 hTert) to a low IR dose (2 Gy) causes a precipitous drop of the normalized MI at 1 h that reflects the prompt activation of the G_2_-checkpoint (Fig. 1A). As mainly cells in G_2_-reach M-phase within the observation time, the effect reflects predominantly the response of cells that were irradiated in G_2_-phase. The checkpoint is maintained for up to 4 h, but cells start re-entering mitosis at later times indicating recovery from the checkpoint that is nearly complete at 8 h.

To examine the contribution of ATR in this response, we treated cells with the specific ATR inhibitor VE-821^93–95^ (to be referred to as ATRi) (Fig. 1A). Strikingly, all stages of the G_2_ checkpoint including initiation, maintenance and recovery are completely abrogated and irradiated cells enter mitosis practically without delay. Similar observations are made with the human lung carcinoma cell line, A549, (Fig. S1A), as well as with several additional cell lines as outlined below. We conclude therefore that the complete control by ATR of the G_2_ checkpoint induced in G_2_ cells exposed to low doses of IR is a rather general response. This observation was unexpected and is novel, as it is widely thought that ATM is the main regulator of the G_2_-checkpoint^4^, and that ATR has a markedly smaller role^11,50,81,90,91^, or that ATR contribution increases only with increasing DNA damage complexity^96^.

To establish that the above results are not confounded by off-target effects of the inhibitor, we carried out similar experiments with GM847-ATRkd cells. These cells are derived from hTert immortalized human fibroblasts, GM847, and express under the control of a Tet-On promoter an ATR fragment with inactivated kinase domain (ATRkd). Incubation with doxycycline (DOX) for 48 h causes expression of ATRkd (Fig. 1B) that exerts a dominant negative effect on ATR^11^.

Before expression of ATRkd, GM847-ATRkd cells exposed to 4 Gy in G_2_ activate the checkpoint as expected and, in line with the results presented above, this activation is fully reversed by treatment with ATRi (Fig. S1B). Notably, expression of ATRkd completely prevents activation of the G_2_-checkpoint (Fig. 1C). ATRi confers under these conditions no additional effect demonstrating that ATR activity is fully suppressed by ATRkd and that ATRi is not conferring detectable off-target effects to cell cycle progression.

To confirm that the above responses are not confounded by dominant negative effects of the inhibited kinase^97^, we also confirmed the central role of ATR in G_2_-checkpoint by depleting the protein using RNAi. In four independent experiments, we observed abrogation of the G_2_-checkpoint after ATR knockdown at levels commensurate to the level of protein depletion, as detected by western blotting. Figures 1D and 1E summarize the results of one such experiment. Collectively, these observations confirm that ATR is absolutely required for the activation of the G_2_-checkpoint in cells exposed to low doses of IR in G_2_-phase, and that ATRi treatment is not associated with side effects that may bias this outcome.

The dominant role of ATR in the G_2_-checkpoint uncovered above raises questions regarding the contribution of ATM, the widely considered, central G_2_-checkpoint and DDR kinase^4^ (see Introduction). Analysis of normalized MI after exposure to a low IR dose (2 Gy) of the ATM mutant AT5BIVA, shows complete loss of the G_2_-checkpoint (Fig. 1F). Similar results have been previously reported^90,91,96^. Unsurprisingly, in this genetic background, treatment with a specific ATM inhibitor (KU55933, to be referred to as ATMi) is ineffective. Notably, also treatment with ATRi is completely ineffective suggesting that the function of ATR on the G_2_-checkpoint at low doses of IR has as prerequisite a functional ATM kinase. Similar results are also obtained in G_2_-irradiated 82-6 hTert cells after treatment with ATMi (Fig. 1A), as well as in A549 cells tested in similar experiments (Fig. S1A). Finally, inhibition of ATM in an ATR deficient background generates results equivalent to those of ATR inhibition alone (Fig. 1C).

Collectively, the above observations suggest that any activation of the G_2_-checkpoint in cells exposed to low doses of IR in G_2_-phase, equally requires the activities of ATM and ATR and not as hitherto believed mainly the activity of ATM with ATR playing a secondary role^91,96^. Based on these results we propose that at low IR doses, ATM and ATR regulate the G_2_-checkpoint as an integrated module in which the two kinases are functionally paired in a fully equivalent and epistatic manner.

This model raises the question as to how the putative ATM/ATR module interphases with the cell cycle to inhibit progression into M-phase of G_2_–irradiated cells. From the known functions of ATM and ATR in checkpoint response^4^, two possibilities can be envisioned: (A) The two kinases in the module signal in parallel, with each kinase independently interfacing and thus regulating cell cycle progression. This reflects the widely held assumption^91,96^. (B). Alternatively, the two kinases in the module may be linked as a linear array, such that one kinase regulates the other, with only the downstream kinase interfacing with and regulating cell cycle progression. Such facets of the ATM/ATR module and their relevance to the regulation of the G_2_-checkpoint are explored next.

### At low IR doses, the ATM/ATR module is organized as a linear array with ATM upstream and with ATR mainly interfacing with the cell cycle engine through Chk1

Mechanistically, the G_2_-checkpoint is implemented by suppressing Cdc25C that activates Cdk1 to drive mitotic entry. ATM and ATR can convey inhibitory signals to Cdc25C through the checkpoint kinases Chk2 and Chk1, respectively^4^. We examined therefore the effect of Chk2 on the G_2_-checkpoint under the low IR dose conditions described above. Treatment of 82-6 hTert cells with the highly specific Chk2 inhibitor (Chk2 inhibitor II, BML-277, to be referred to as Chk2i) fails to generate detectable effects on the induction of the checkpoint and has only a small effect on its recovery (Fig. 1G), as already reported before^28,30,33,91,98^. Similar results are obtained with A549 cells (Fig. S1D).

On the other hand, UCN-01, a specific inhibitor of Chk1 (to be referred to as Chk1i) causes a marked, albeit incomplete, suppression of the G_2_-checkpoint in 82-6 hTert and A549 cells at the indicated doses (Figs 1G and S1D), suggesting that the tight control of the G_2_–checkpoint exerted by ATR is mediated by the activation predominantly, but evidently not entirely, of Chk1^91^. We conclude that at low doses of IR, ATR is downstream of ATM and connects with the cell cycle machinery through Chk1.

**Figure 2:**
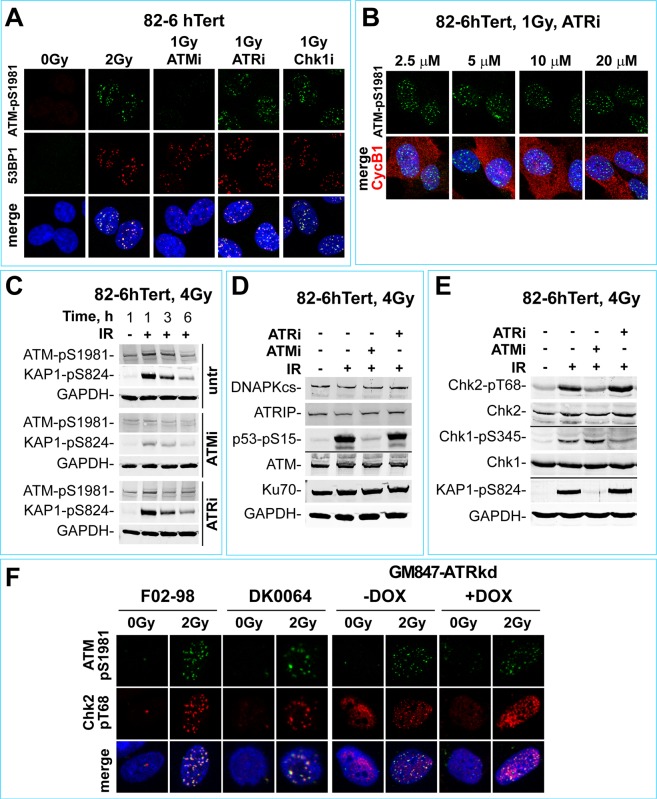
ATR inhibition leaves ATM signaling intact. (**A**) IF of ATM-pS1981 (green) and 53BP1 (red) foci in 82-6 hTert cells 1 h after exposure to 1 Gy. Blue: DAPI staining. (**B**) IF analysis of ATM-pS1981 foci (green) in late-S/G_2_ (Cyclin B1 positive, red) 82-6 hTert cells exposed to 1 Gy, 1 h after treatment with the indicated concentrations of ATRi. (**C**) Western blot analysis of 82-6 hTert cells treated with ATRi or ATMi for 1 h and exposed to 0 or 4 Gy under continuous incubation with the inhibitors. GAPDH serves as a loading control. (**D**,**E**) Western blot analysis monitoring DNA-PKcs, ATM, ATRIP and Ku70, as well as activation of the downstream PIKK targets p53-pS15, KAP1-pS824, Chk2-pT68 and Chk1-pS345 in 82-6 hTert cells treated with ATRi and ATMi after exposure to 4 Gy. GAPDH, as well as non-phosphorylated Chk1 and Chk2 serve as loading controls. (**F**) IF of ATM-pS1981 (green) and Chk2-pT68 (red) 30 min after exposure to 2 Gy of the Seckel syndrome cell lines, F02-98 and DK0064, as well as of GM847-ATRkd cells tested in the absence (−DOX) or presence (+DOX) of DOX.

The proposed organization of ATM and ATR in a module raises the question as to whether genetic or chemical inactivation of ATR somehow feeds back to also suppress ATM activation and thus also its contribution to the checkpoint. To investigate this possibility, we examined ATM activation under conditions of suppressed ATR activity. Exposure of 82-6 hTert cells to IR induces DSBs as indicated by the formation of 53BP1 foci and activates ATM: as shown by the generation of phospho-ATM (ATM-pS1981), detectable by immunofluorescence (IF) (Fig. 2A,B) or by western blot analysis (WB) (Fig. 2C–E). ATM activation is also documented by the phosphorylation of ATM downstream targets: KAP1 on Serine 824 (KAP1-pS824) (Fig. 2C), p53 on Serine 15 (p53-pS15) (Fig. 2D) and Chk2 on Threonine 68 (Chk2-pT68) (Figs 2E and S2A for A549 cells). All these facets of ATM activation and signaling are strongly suppressed by ATMi, but remain unaffected by ATRi (Fig. 2A–E) or Chk1i (Fig. 2A). Vice versa, phosphorylation of Chk1 on Serine 345 is suppressed by ATRi but remains strong after treatment with ATMi (Fig. 2E). Very similar results are also obtained with A549 cells (Fig. S2B,C). Moreover, cells derived from patients with Seckel syndrome, a hereditary form of microcephaly dwarfism caused by an ATR pre-mRNA splicing mutation that reduces ATR protein levels^99^, F02-98 and DK0064, and DOX-treated GM847-ATRkd cells (Fig. 2F), show normal ATM and Chk2 activation.

Collectively, the above results show that inhibition of ATR, or of its downstream effector Chk1, does not interfere with ATM activation or signaling. Yet, despite strong activation of several of its facets, in the absence of ATR activity, ATM is unable to sustain the checkpoint by directly interfacing with the cell cycle either through activated p53 (Fig. 2D), or through Chk2 (Fig. 2E,F).

These observations further support the notion of a linear functional integration of ATM and ATR in a module, with ATR downstream of ATM and with ATR exclusively connected to the cell cycle engine. Activation of ATM in this module relies on upstream inputs and remains unaffected by ATR inhibition. Also facets of ATR activation remain unaffected after incubation with ATMi, as indicated by the robust phosphorylation of Chk1 after inhibition of ATM (Fig. 2D). Next we analyze ATR activation by the induction of resection at DSBs and examine the role of ATM in this activation.

### At low IR doses, ATR is activated by resection, epistatically coordinated by the ATM/ATR module

The requirement for ATR in the G_2_-checkpoint makes resection, the prerequisite of ATR activation at DSBs^4^, also a checkpoint requirement. Therefore, we utilized IF to measure resection by analyzing RPA retention at the chromatin as a function of time after IR. To specifically analyze cells in G_2_-phase and generate results directly comparable to those discussed above for the checkpoint, we labeled cells in S-phase by incubating cultures with EdU for 30 min just before IR. In thus labelled cell populations, we quantitated RPA signal specifically in G_2_-phase cells, identified by DAPI-signal analysis. The parallel exclusion in this analysis of EdU positive cells, ensured that cells in late-S, as well as S-phase cells that entered G_2_ during the post-irradiation period of observation, were not confounding the results.

**Figure 3:**
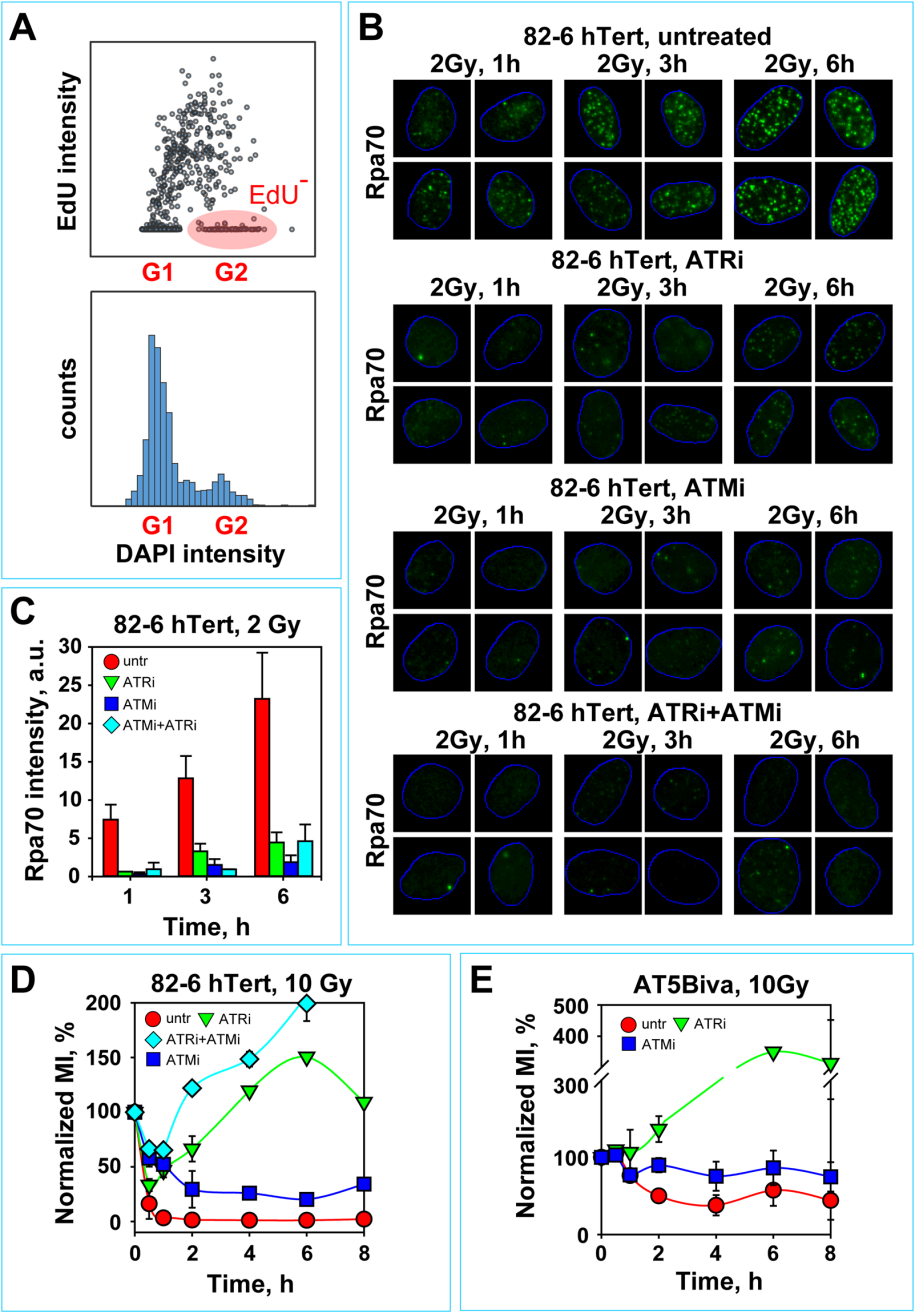
ATM and ATR are both essential for DNA end-resection in G_2_–phase cells exposed to low IR doses. (**A**) Upper panel: Representative dot plot of EdU and DAPI signals obtained by automated high-throughput IF image analysis (Metasystems) of 1600 exponentially growing 82-6 hTert cells; this type of plot is used to define gates for selecting EdU negative (EdU^−^), G_2_-phase cells (red ellipse), to carry out the resection analysis described in the following panels. Lower panel: Cell cycle distribution of the analyzed cell population as determined by the intensity of the DAPI signal. (**B**) Representative images showing Rpa70 signal, a measure of resection at DSBs, as a function of time in 82-6 hTert cells exposed to 2 Gy specifically in G_2_-phase and incubated in the absence or presence of ATMi and/or ATRi. (**C**) Quantitative analysis of integrated Rpa70 signal intensities in G_2_-irradiated 82-6 hTert cells at different times after exposure to 2 Gy and incubated with the indicated PIKK inhibitors. (**D**) Normalized MI as a function of time in 82-6 hTert cells pre-treated with ATMi or ATRi and exposed to 10 Gy. Other details as in Fig. 1A. (**E**) As in (**D**), but for AT5Biva cells. The data shown represent the mean and standard deviation from 3 independent experiments.

Figure 3A outlines the procedure used for the analysis of resection and the gates adopted for measuring RPA signal specifically in cells irradiated in G_2_ (gate marked in red). Figure 3B shows representative images of 82-6 hTert cells exposed to 0 or 2 Gy and stained for RPA at different times after IR. Figure 3C shows the results obtained by quantifying integral RPA signal in 100–150 G_2_-phase cells. The plotted values were calculated by subtracting the corresponding background signal detected in the same cohort of non-irradiated cells. We selected total RPA intensity per cell as a parameter in our analysis, reasoning that it reflects the level of ATR activation better than scoring of individual foci. Indeed, a reduction in total RPA signal reflects a reduction in ATR activation more accurately than a reduction in foci numbers. In addition, this form of analysis is equivalent to integral measurements carried out using flow cytometry in similarly processed cells after exposure to high doses of IR (see below).

It is evident (Fig. 3B,C) that with progressing incubation time after IR the level of resection increases in G_2_–irradiated cells, in line with the ATR activation required to sustain the checkpoint (Fig. 1A). Treatment with ATMi suppresses, as expected, resection practically completely. Suppression of resection by ATM inhibition will also suppress ATR activation in line with the checkpoint results presented above. Strikingly, also incubation with ATRi suppresses resection to a degree comparable to that observed after treatment with ATMi (Fig. 3B,C). Combined treatment with ATRi plus ATMi only has a small additive effect.

We conclude that the postulated ATM/ATR module also regulates resection, utilizing similar epistatic and functional interconnections between ATM and ATR as in checkpoint regulation (see above). The practically complete regulation of resection by ATRi is novel, as hitherto ATM was considered the major regulator of resection^4^, and suggests a feedback loop from ATR to ATM in this regulation. Strikingly, this feedback inhibition does not involve other canonical signaling functions of ATM, because as we showed above (Fig. 2A–F), these remain intact after inhibition of ATR.

### High IR doses uncouple the function of ATM from ATR and enable independent ATM or ATR signaling to the cell cycle machinery

Fortuitously noted deviations in the responses described above when different ranges of doses were used, as well as the results of ongoing studies on DSB repair pathway choice in G_2_, prompted the systematic analysis of relevant endpoints at higher IR doses. First, we examined the role of ATR in G_2_-checkpoint in 82-6 hTert cells exposed to 10 Gy. Figure 3D shows that this high IR dose initiates a strong checkpoint that prevents any cells from entering mitosis during the period of observation. Remarkably, although treatment with ATRi alleviates this response, more than 70% of cells actually initiate a checkpoint, from which they recover 4 h later. Strikingly, under conditions of ATM inhibition the G_2_-checkpoint is also initiated, albeit slightly later and to a lesser degree, but once mounted it persists for the period of observation. Thus, at high IR doses, and in stark contrast to the results obtained at low IR doses, cells are able to mount sizeable checkpoint responses in an ATR deficient, and even more so, in an ATM deficient background. The response described here after exposure to high IR doses has been frequently reported in the literature and is at least partly responsible for the widely held view that ATR is only contributing to the activation of an ATM dependent checkpoint in ways that depend on cell line and form of DNA damage induced^90,91,96^.

Notably, the residual G_2_-checkpoint observed in ATRi- or ATMi-treated cells is further suppressed by treatment with the complementary inhibitor, an effect that we and others have reported earlier for ATM deficient cells^80,81^. Thus, only a weak and very transient checkpoint that quickly overshoots is noted in cells treated with combined ATRi + ATMi (Fig. 3D; see also below). We conclude that at high IR doses, in the absence of ATR activity, a reduced-intensity ATM-dependent checkpoint develops and vice-versa that in the absence of ATM a reduced intensity ATR-dependent checkpoint develops. Cells have only very limited potential for mounting a checkpoint response in the combined absence of ATM and ATR activities even after exposure to high doses of IR.

The responses observed in wild-type cells using inhibitors are also confirmed with AT cells. AT5BIVA cells (Fig. 3E) mount a pronounced and persistent G_2_-checkpoint after exposure to 10 Gy^80,81^. Treatment with ATRi fully reverses the checkpoint, to the extent that 2 h after IR mitotic indices higher than those of non-irradiated cells are measured, a response also observed in Fig. 3D.

The high IR-dose results uncover two key regulatory aspects of the mechanism of checkpoint activation that contrast sharply the regulation characterized at low IR doses. These include the observation that at high IR doses, inhibition of one kinase fails to fully suppress the contribution of the other towards the checkpoint, as well as the observation that at high IR doses both kinases independently interphase with the cell cycle engine and cause cell cycle arrest. Evidently, in the high dose range, the modular pairing of ATM and ATR changes in ways that allow independent outputs to the cell cycle machinery from either kinase individually.

### High IR doses uncouple the functions of ATM and ATR in DNA end resection and generate conditions permissive to independent regulatory contributions

**Figure 4:**
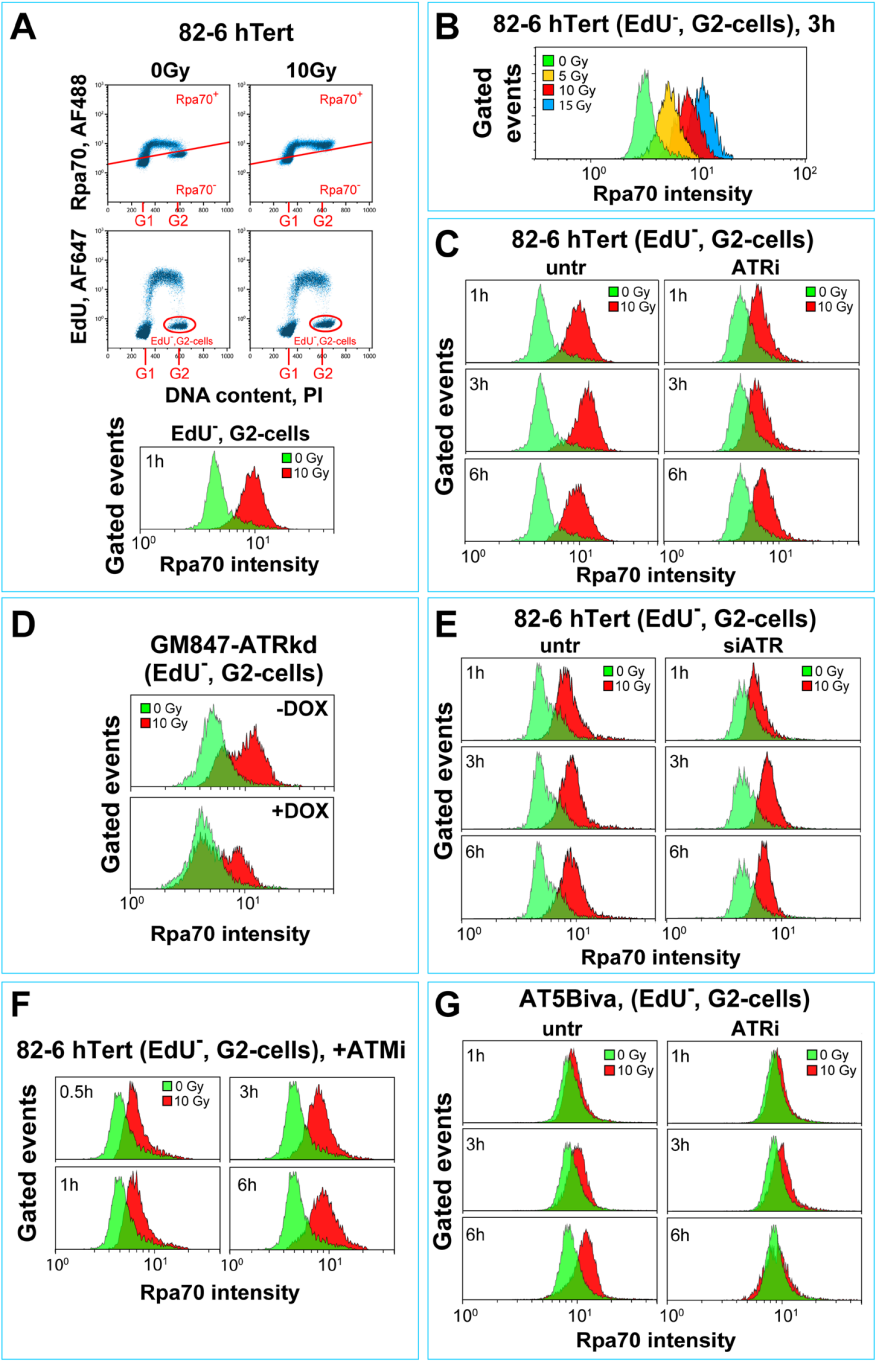
ATM and ATR independently regulate DNA end-resection in G_2_-phase cells exposed to high IR doses. (**A**) Outline of the three-parametric flow cytometry analysis employed to quantitate DNA end-resection in G_2_-irradiated cells exposed to high IR doses. Upper panels: Dot plots showing Rpa70 *versus* PI signals. Middle panels: Dot plots showing EdU *versus* PI signals. Shown in these panels are also the gates applied for quantitating resection at different times after IR in G_2_-cells (EdU^−^). Lower panels: Histograms of Rpa70 signal detected in non-irradiated (0 Gy, green) and irradiated, (10 Gy, red) G_2_-phase cells. (**B**) Representative histograms of Rpa70 signal intensity in G_2_–phase, 82-6 hTert cells exposed to increasing IR doses (1 h) as indicated. (**C**) Histograms of Rpa70 signal intensity as a function of time in G_2_-phase, 82-6 hTert cells exposed to 10 Gy in the presence or absence of ATRi. (**D**) Histograms of Rpa70 signal intensity at 1 h after exposure of GM847-ATRkd cells to 10 Gy in G_2_-phase following pretreatment (+DOX) or not (−DOX) with DOX. (**E**) Histograms of Rpa70 signal intensity as a function of time, in 82-6 hTert cells exposed to 10 Gy in G_2_-phase 24 h after transfection with siRNA targeting ATR, and in untreated controls. (**F**) Histograms of Rpa70 signal intensity as a function of time in G_2_-phase, 82-6 hTert cells irradiated with 10 Gy in the presence or absence of ATMi. (**G**) As in C, but for AT5Biva cells.

To study the roles of ATM and ATR in the regulation of resection at high IR doses, in a cell cycle specific manner, we adapted a previously published, quantitative flow cytometry-based method^100^. In this method, cultures are incubated, as for IF, with EdU to label cells in S-phase and resection is measured by staining of chromatin-bound RPA in EdU negative, G_2_-phase cells, identified by PI co-staining of DNA (Fig. 4A). Resection (i.e. Rpa70 signal) is analyzed as a function of time after IR, always in G_2_, exclusively for EdU negative (EdU^−^) cells. Events in this G_2_-compartment represent cells irradiated and still residing at the time of analysis in G_2_-phase. Figure 4A shows representative data and the gates applied to quantitate RPA, EdU and PI signals 1 h after exposing 82-6 hTert cells to 0 or 10 Gy (upper panels). RPA signal intensity distribution in the defined gates is shown in the lower panel. The marked RPA signal-increase noted after IR, reflects the robust resection that is induced at DSBs in G_2_ cells of this cell line.

Systematic signal analysis in EdU^−^, 82-6 hTert cells in the G_2_-compartment shows that quantitation of resection is possible in a range of doses between 5–15 Gy (Fig. 4B). Figure 4C shows that EdU^−^ cells exposed to 10 Gy show robust resection that is well advanced at 1 h after IR, reaches a maximum at 3 h and begins to recover at later times. Notably, at this dose and in contrast to the results shown for low doses in Fig. 3C, incubation with ATRi suppresses resection by only about 50% (Fig. 4C, right panels). This partial suppression of resection is ATRi-concentration dependent reaching a maximum at 5–10 μM, at 1 h or 3 h after IR (Fig. S3A). Notably, ATRi has no effect on RPA signal intensity of the G_2_ compartment in the absence of IR.

Similar results are obtained with G_2_-irradiated A549 cells (Fig. S3B), although here the degree of resection in untreated cells is much lower than in 82-6 hTert cells and as a result the effect of ATRi appears complete. Furthermore, G_2_-irradiated GM847-ATRkd cells show in the absence of DOX significant resection that is partly suppressed after expression of ATRkd (DOX) (Fig. 4D). A reduction in resection is also observed upon knockdown of ATR (Fig. 4E). Thus, at high IR doses, inhibition of ATR, using either genetic approaches or inhibitors, suppresses resection only partially. This result is reminiscent to the partial suppression also observed for the G_2_-checkpoint in the same range of high IR doses.

After exposure to high IR doses, also inhibition of ATM causes a clear but only partial suppression of resection in G_2_ 82-6 hTert cells (Fig. 4F). A similar response is also observed in AT5BIVA cells (Fig. 4G, left panels), as well as in AT hTert fibroblasts (Fig. S3C).

**Figure 5:**
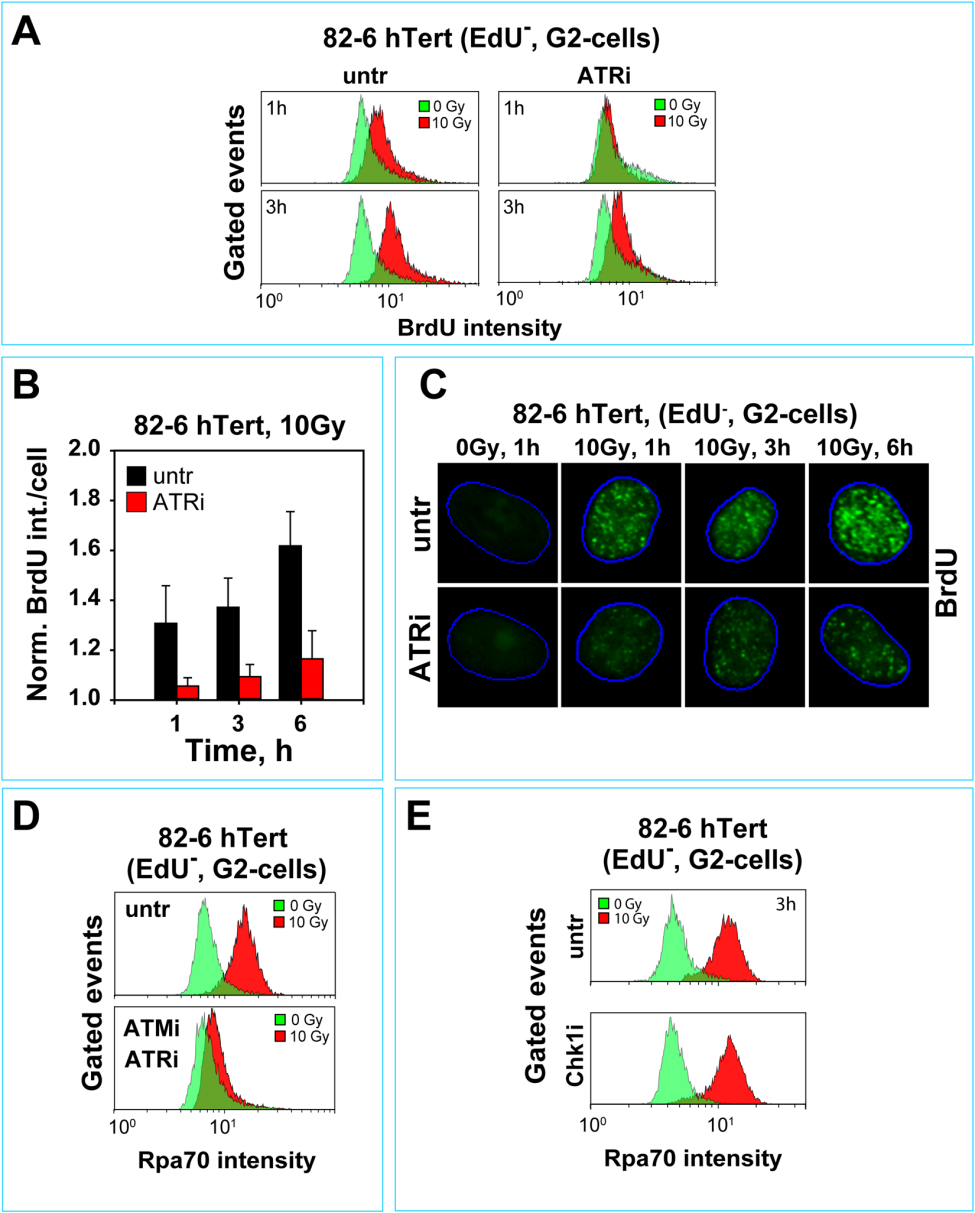
Analysis of DNA end-resection by BrdU staining in G_2_-phase cells. (**A**) BrdU signal intensity under non-denaturing conditions in EdU^−^, G_2_-phase, 82-6 hTert cells, exposed to 10 Gy in the presence or absence of ATRi. Cells were pulse-labeled with EdU just before IR to identify cells irradiated in G_2_-phase. Only EdU^−^, G_2_-phase cells were included in the analysis. Other details of analysis are as in Fig. 4A. (**B**) IF based quantification analysis of BrdU signal as a function of time in 82-6 hTert cells analyzed under non-denaturing conditions after exposure to 10 Gy in the presence or absence of ATRi. **(C)** Representative IF images of G_2_-phase, 82-6 hTert cells captured during the quantification analysis shown in (**C**). (**D**) Histograms of Rpa70 signal intensity in 82-6 hTert cells, 3 h, after exposure to 10 Gy in G_2_-phase and incubation in the combined presence of ATMi and ATRi. (**E**) As in D for cells incubated with Chk1i.

To validate our G_2_-resection results using an alternative approach, we grew 82-6 hTert cells with BrdU and used flow cytometry or IF to quantitate resection-generated ssDNA, by staining with antibodies against BrdU under non-denaturating DNA conditions. Figure 5A shows flow cytometry results confirming that after exposure to high IR doses, ATRi only partly suppresses resection; similar results are obtained with immunofluorescence analysis (Fig. 5B,C).

Since ATM inhibition on an ATR deficient background suppresses the G_2_-checkpoint almost completely, we also tested the effect of combined inhibition of ATM and ATR on resection. Figure 5D shows that inhibition of both kinases causes nearly complete suppression of resection. Also inhibition of ATR on an ATM deficient background fully suppresses resection (Fig. 4G, S3C, right panels).

Strikingly, in contrast to the strong effect on resection observed after treatment with ATRi, incubation with Chk1i leaves resection unchanged, both in 82-6 hTert (Fig. 5E), as well as in A549 cells (not shown) irradiated in the G_2_-phase of the cell cycle. The above results extend the observations on G_2_-checkpoint regulation to the regulation of DSB resection. Because DNA-PKcs is the PI3K thought to engage DSBs well before ATM or ATR, we investigated next possible crosstalk of DNA-PKcs with the ATM/ATR module, and contributions to the regulation of checkpoint and resection.

### DNA-PKcs suppresses an ATM/ATR dependent G_2_ checkpoint hyperactivation

**Figure 6:**
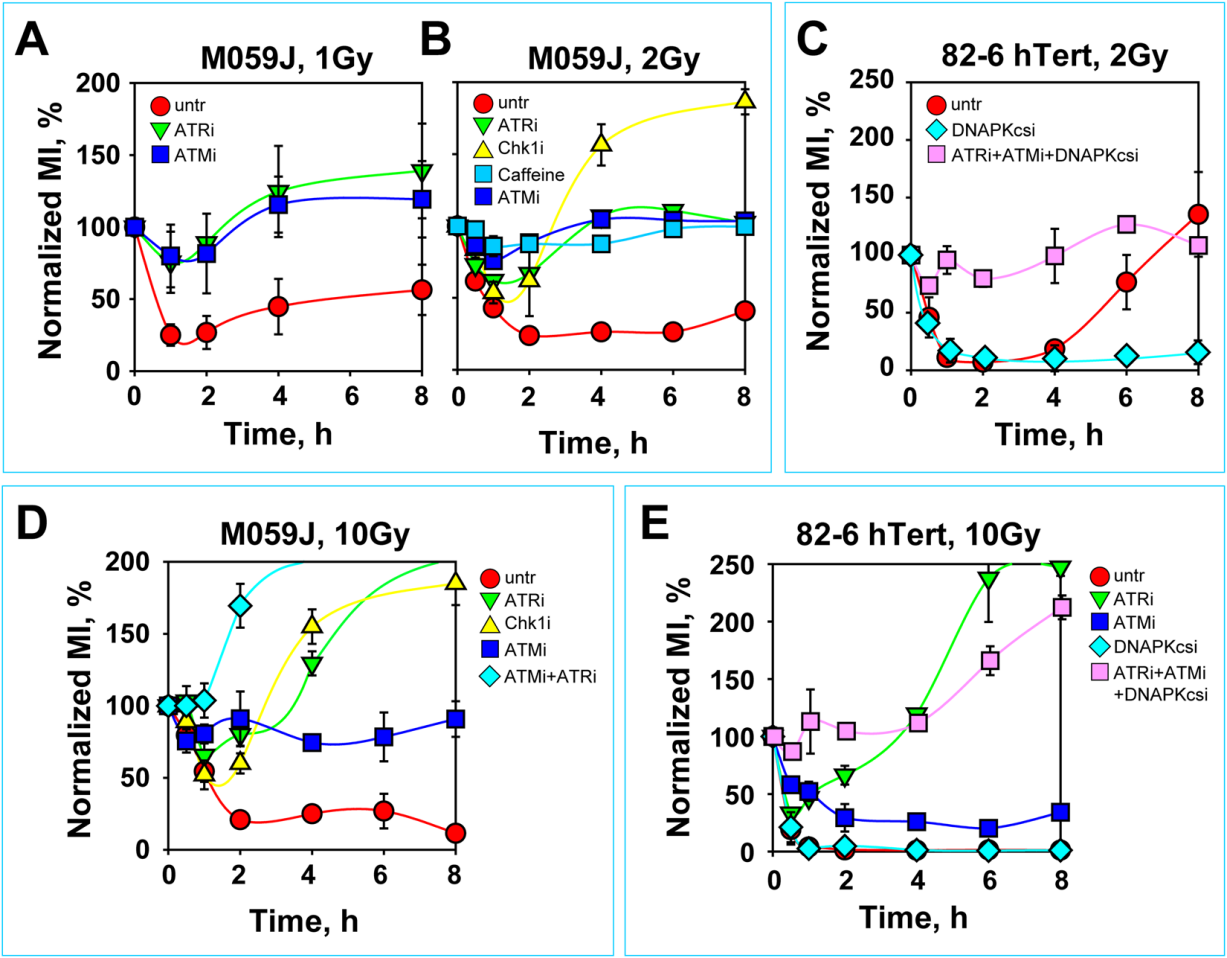
DNA-PKcs deficiency causes hyper-activation of the G_2_-checkpoint. (**A,B**) Normalized MI as a function of time in M059J cells treated with the indicated PIKK inhibitors and exposed to 1 or 2 Gy. The values of MI used for normalization in different experiments were: MI_untr_ = (0.91–2.59%), MI_ATRi_ = (1.31–3.13%), MI_ATMi_ = (1.34–2.69%). (**C**) Normalized MI as a function of time in 82–6 hTert cells treated with the indicated combinations of PIKK inhibitors after exposure to 2 Gy. The values of MI used for normalization in different experiments were: MI_untr_ = (1.78–3.44%), MI_DNAPKcsi_ = (2.87–6.14%), MI_ATRi/ATMi/DNAPKcsi_ = (0.89–3.03%). (**D**) Normalized MI as a function of time in M059J cells treated with the indicated PIKK inhibitors and exposed to 10 Gy. Other details as in A. (**E**) Normalized MI as a function of time in 82-6 hTert cells treated with the indicated combinations of PIKK inhibitors and exposed to 10 Gy. Other details as in (**C**).

Exposure in G_2_ of DNA-PKcs deficient M059J (Fig. 6A,B) and HCT116 *DNA PKcs*^−/−^ cells (Fig. S4A) to low IR doses (1–2 Gy) causes strong and persistent reduction in the normalized MI as compared to DNA-PKcs proficient M059K (Fig. S4B), or wild-type HCT116 cells (Fig. S4C). This documents a hyperactivation of the G_2_-checkpoint as a consequence of the DNA-PKcs defect (see Fig. S4D for DNA-PKcs expression in M059K and M059J cells). Notably, this hyperactive checkpoint is sustained by the functionally integrated ATM/ATR module, as inhibition of either kinase completely abrogates its induction, particularly when M059J cells are exposed to 1 Gy (Fig. 6A). Also cells exposed to 2 Gy show similar trends, although the alleviation of the checkpoint response following incubation with a single kinase inhibitor is slightly less pronounced (Fig. 6B) suggesting an early transition to high IR dose response. As expected, inhibition of both ATM and ATR by incubation with caffeine causes a practically complete suppression of the checkpoint response both in M059J cells (Fig. 6B), as well as in HCT116 *DNA PKcs*^−/−^ (Fig. S4A) and M059K cells (Fig. S4B). Several facets of checkpoint activation including stabilization of p53, formation of KAP-pS824 and Chk2-pS68 (Fig. S4E), as well as the activation of ATM despite inhibition of ATR (Fig. S4F), remain in M059K and M059J cells as described above for 82-6 hTert and A549 cells.

82-6 hTert cells respond after treatment with the specific DNA-PKcs inhibitor, NU7441 (to be referred here as DNA-PKi) as DNA-PKcs deficient cells (Fig. 6C). Notably, also in this setting combined additional inhibition of ATM and ATR fully abrogates the checkpoint (Fig. 6C).

Exposure of M059J cells to high IR doses (10 Gy) initiates as expected a strong checkpoint that shows no signs of recovery during the observation period (Fig. 6D). Incubation with ATRi suppresses now only partly the checkpoint, as already shown for DNA-PKcs proficient cells. Also incubation with ATMi leaves a residual checkpoint, which in this cell line is marginal, probably as a consequence of reduced ATM levels (Fig. S4D)^72^. Similar results are obtained after exposure of HCT116 *DNA PKcs*^−/−^ cells to 10 Gy, and in this case the effect of ATMi is similar to that observed in wild-type cells exposed to high IR doses (Fig. S4G). Notably, at high IR doses, even in the DNA-PKcs deficient genetic background, combined inhibition of ATM and ATR causes a practically complete abrogation of the checkpoint. A similarly impressive response is observed in 82-6 hTert cells, where exposure to 10 Gy causes a robust checkpoint after inhibition of DNA-PKcs, whereas combined inhibition of all three kinases causes a complete abrogation of the checkpoint (Fig. 6E). Communication of the checkpoint response to the cell cycle engine is through Chk1, although the dependence is here again only partial (Fig. 6B,D and S4A,G).

Collectively, the above results show that the functionality and intensity of pairing of the ATM/ATR module remains intact in a DNA-PKcs deficient background. Also, the IR-dose-dependent function of the ATM/ATR module can be clearly observed in DNA-PKcs deficient cells, albeit slightly earlier, as compared to DNA-PKcs proficient cells. We conclude that normally DNA-PKcs suppresses processes causing ATM/ATR-induced hyperactivation of the G_2_-checkpoint, as previously reported for the intra-S-phase checkpoint^101^ and more recently for additional endpoints^73,74^.

### DNA-PKcs suppresses ATM/ATR regulated resection

**Figure 7:**
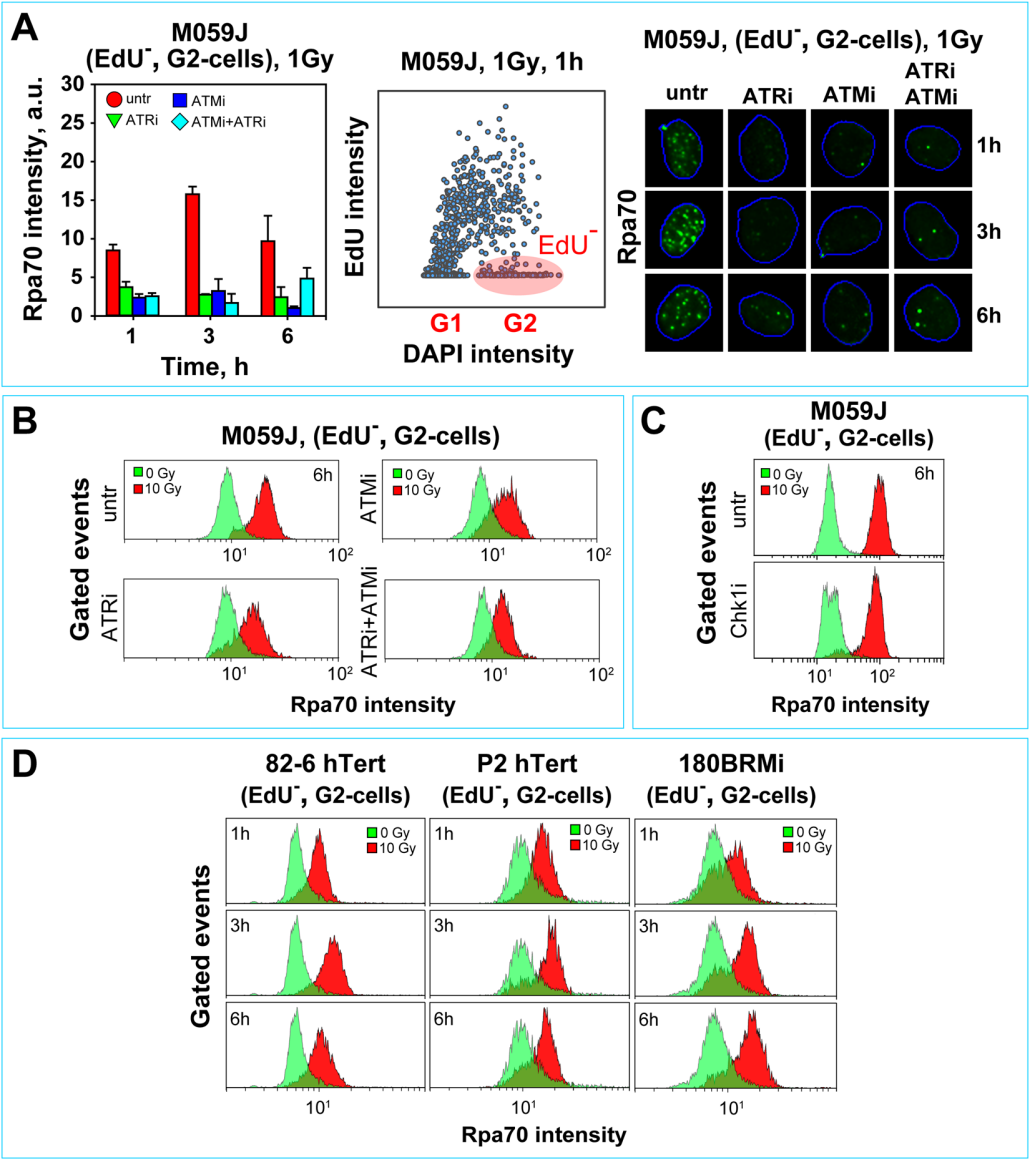
DNA-PKcs deficiency causes kinase specific hyper-resection in G_2_-phase irradiated cells. (**A**) Left panel: Quantitative analysis of integrated Rpa70 signal intensity in G_2_-irradiated M059J cells at different times after exposure to 1 Gy and incubation with the indicated PIKK inhibitors. Middle panel: Representative dot plot of EdU and DAPI signals obtained by automated high-throughput IF image analysis (Metasystems) of 1600 exponentially growing M059J cells; this type of plot is used to define gates for selecting EdU negative (EdU^−^), G_2_-phase cells (red ellipse), to carry out the resection analysis described in the left panel and in the following experiments. Right panels: Representative images captured during quantitative analysis of integrated Rpa70 signal intensity shown in (**A**). (**B**) Histograms of Rpa70 signal intensity in G_2_-phase, M059J cells, 6 h after exposure to 10 Gy in the presence or absence of ATRi, ATMi, or ATM + ATRi. (**C**) As in B, but for cells treated with Chk1i. (**D**) Histograms of Rpa70 signal intensity as a function of time in 82-6 hTert, XLF deficient P2 hTert, and Lig4 deficient 180BRMi cells, exposed to 10 Gy in G_2_-phase.

Exposure of DNA-PKcs deficient M059J cells to the low IR dose of 1 Gy induces strong resection (see Fig. 7A–C) that is nearly completely inhibited after incubation with either ATMi or ATRi (as well as ATMi + ATRi) confirming the integrated, paired function of the ATM/ATR module in resection, also in a DNA-PKcs deficient background (Fig. 7A).

At high IR doses (10 Gy), M059J cells show markedly enhanced resection (Fig. 7B) as compared to M059K cells (Fig. S5A). Under these conditions, ATRi or ATMi reduce resection only partly (Fig. 7B), while Chk1i has no effect (Fig. 7C). Similar observations are made in M059K cells (Fig. S5A). Notably, even in a DNA-PKcs deficient genetic background, combined treatment with ATMi and ATRi strongly suppresses resection induced by high IR doses (Fig. 7B). Similar results are obtained in irradiated (10 Gy) HCT116 DNA-PKcs^−/−^ cells, and here again combined treatment with ATMi plus ATRi abrogates resection almost completely (Fig. S5B). Resection is also almost entirely suppressed in 82-6 hTert cells exposed to 10 Gy and treated with ATMi + ATRi + DNA-PKcsi (Fig. S5C).

As possible explanation for the hyperresection observed in DNA-PKcs deficient cells, one could invoke the associated inhibition of c-NHEJ that may non-specifically allow more DSBs to be processed by resection-dependent repair pathways, HRR, alt-EJ or SSA. To test this possibility, we carried out similar experiments evaluating resection in mutants of c-NHEJ with defects in repair pathway components other than DNA-PKcs. We reasoned that if a general inhibition of c-NHEJ, rather than a specific defect in DNA-PKcs, causes the enhanced resection, then all c-NHEJ mutants should show this response. For experiments, we selected mutant human fibroblasts deficient in XLF or Lig4, because they display DSB repair defects stronger than DNA-PKcs deficient mutants. Therefore, if a general inhibition of c-NHEJ underpinned the hyperresection of DNA-PKcs deficient cells, we would expect these mutants to show an even stronger enhancement in resection. Figure 7D shows that, contrary to these expectations, P2 hTert human fibroblasts deficient in XLF, and 180BRMi immortalized human fibroblasts deficient in Lig4, show resection in G_2_ after exposure to 10 Gy that is comparable or lower than that of 82-6 hTert cells (Fig. 7D), and which is uniformly reduced after treatment with ATRi (Fig. S5D).

## Discussion

While the individual functions of ATM and ATR in DDR are well-characterized^4^, aspects of functional overlap and crosstalk are still emerging. The defining characteristic underpinning functional interactions between these kinases is their central involvement in recognizing, signaling from and ultimately regulating processing of DSBs. Specifically, ATM-dependent resection at a DSB facilitates ATR activation^4,33,48–50,102,103^ and mediates an activity switch from ATM to ATR^79^. Consistently, depletion, or inactivation of Mre11, Exo1, and Dna2 leads to diminished ATR responses to DSBs^48–50,104,105^. In addition to this form of crosstalk, functional complementation has also been also reported, with ATR enforcing a G_2_-checkpoint in ATM-deficient cells^80,81,91,96^.

Here we provide the first evidence that the functional interactions of ATM and ATR go well beyond occasional crosstalk or functional complementation. We demonstrate a complete functional coupling between the two kinases in the regulation of DSB resection and G_2_-checkpoint activation in cells sustaining small loads of DSBs through exposure to low IR doses, specifically in the G_2_-phase of the cell cycle. Under these conditions, ATM and ATR are equally required for the regulation of these key aspects of DDR, while functioning in a fully epistatic manner. Our results uncover a hitherto unreported functional coupling between ATM and ATR in a module with internal directional crosstalk-characteristics that remain to be elucidated, and connections to the cell cycle machinery largely through ATR to Chk1 signaling^106^. Such a modular integration raises ATR’s function and importance for these endpoints to the same level as that of ATM and suggests that the classical, ATM-centered regulatory organization of the G_2_-checkpoint needs revision^107^.

The fundamental requirement for resection in ATR activation, implicates resection also in the activation of the G_2_-checkpoint and predictably, our results show G_2_-checkpoint activation when resection is also detectable. Strikingly though, ATR is not only passively responding to resection, but is also actively regulating this process in the low dose region. However, regulation of resection by ATR is not via Chk1, as in the case of checkpoint regulation, but via Chk1-independent branches of the ATR regulatory network. The role for ATR in resection may not be surprising since ATR is known to phosphorylate CtIP^108^, but we anticipate additional regulatory branches that remain to be elucidated^105^.

Resection at a DSBs is an important determinant of DSB-repair-pathway choice favoring HRR, SSA, and alt-EJ^19,87,109^. The linkage between G_2_-checkpoint activation and resection implies that DSBs induced by low IR doses and processed by c-NHEJ will not activate the G_2_-checkpoint and will therefore not directly benefit from cell cycle delays. Indeed, c-NHEJ is not linked to ATR signaling and the G_2_-checkpoint in a number of experimental settings^110–113^. Such selective activation of the G_2_-checkpoint by resected DSBs that excludes DSBs processed by c-NHEJ, offers mechanistic explanation why this checkpoint appears “leaky” in some settings^114^.

Highly relevant for our understanding of DDR is our novel observation that the function of the ATM/ATR module changes as the load of DSBs in the genome of G_2_-phase cells increases - both for resection, as well as for checkpoint activation. Indeed, the tight coupling and epistasis between ATM and ATR relax at high IR doses, and under these conditions each kinase independently, albeit only partly, regulates resection and G_2_-checkpoint activation. Such “contributing” responses from each kinase individually, have been repeatedly reported in the past for these endpoints and have been discussed above.

The observation of IR dose-dependent adaptations in the molecular underpinnings of DDR, strengthens the recognition that the load of DNA damage, in all its possible forms, is closely monitored and used to appropriately adjust the elicited cellular responses^88,89,115^. This is highly relevant because in the analysis of cellular responses to IR, artificial, frequently purpose-adjusted but rarely mechanistically justified, limits are defined between cellular effects generated by low *versus* high IR doses, and high dose effects are frequently discounted as “non-physiological”.

Our experiments contribute to this debate by demonstrating that, indeed, there are important mechanistic shifts in the responses cells mount after exposure to low *versus* high IR doses. However, our work also demonstrates that the ability of a cell to adapt to large increases in DSB load is ingrained in its physiology, serving the maintenance of its genome. Therefore, high dose responses ought to be regarded as equally “physiological” to low dose responses. Consequently, research in the field should focus on understanding high and low -IR -dose responses, as well as the molecular basis of mechanistic shifts occurring with increasing dose. Such dose dependent mechanistic shifts should also be clearly separated from effects resulting from overexposures that may destroy the DDR system itself. Of course, fields such as human radiation protection, will naturally emphasize low dose effects, as defined by the range of doses humans are likely to encounter on earth and possibly in space, but should not remain ignorant to high -dose- response adaptations.

The presence in cells of higher eukaryotes of mechanisms handling DNA damage induced by doses of many Gy of IR generates an evolutionary conundrum, as doses of this magnitude were not present long enough to generate the required evolutionary pressure. It is likely therefore that their evolution benefited not only the stability of very large genomes under conditions of modest DSB induction, but also genome sculpting for speciation and genome rescue in the face of catastrophic events such as chromothripsis^116^.

The last significant contribution of the experiments reported here is the direct crosstalk of DNA-PKcs with the ATM/ATR module, detected as hyper-resection and G_2_-checkpoint hyperactivation in DNA-PKcs deficient cells. Notably, this effect is not, as one might expect at first, the consequence of a non-specific c-NHEJ deficiency, as other c-NHEJ mutants fail to show even slight hints of a similar response. Such crosstalk is mechanistically supported by the recent report that DNA-PKcs exerts strong negative regulation on ATM through phosphorylation at multiple sites^73^, and by the observation that DNA-PK stimulates the MRN complex and CtIP for efficient endonucleolytic processing of DNA ends under physiological conditions^117^.

We conclude therefore that DNA-PKcs has a role in DSB repair pathway choice, which is more pro-active than the hitherto postulated, passive, autophosphorylation-mediated release of captured DNA ends^6^, to modes of processing alternative to c-NHEJ. We propose that DNA-PKcs and Ku by virtue of their extremely high cellular abundance and phenomenal affinity for DNA ends, will capture all DNA ends generated in a G_2_-irradiated cell, even at very high IR doses, and will actively coordinate all further processing. We hypothesize that for such coordination of further processing, DNA-PK integrates inputs in the chromatin environment that remain to be characterized, but which include DNA damage load, and depending on outcome, directs a fraction of DSBs to c-NHEJ, while shunting the rest to resection-dependent pathways of processing. In this model, DNA-PKcs remains a central regulatory component in the repair, also for DSBs processed by resection-dependent pathways. Ultimate goal is maximum preservation of the genome under the prevailing conditions.

Notably, DNA-PKcs may not be affecting the internal underpinnings of the ATM/ATR module, but may only regulate the amplitude of its response, as indicated by the preservation of the IR dose-dependence established in its function in DNA-PKcs proficient cells. Since in the absence of ATM/ATR, DNA-PKcs is unable to implement a checkpoint response, we conclude that under the conditions examined here, DNA-PKcs lacks direct links to the cell cycle engine.

Defining the molecular underpinnings of the cross-regulatory interactions between DNA-PKcs, on the one hand, and ATM/ATR, on the other hand, is a promising area for future mechanistic investigations. Our working hypothesis is that DSBs always activate DNA-PKcs first, which then depending on chromatin context and other parameters, either process the break, or actively initiates through interactions with ATM resection processes. Such resection activates in turn ATR, which together with ATM regulates further processing and checkpoint response. Critical at this stage is the crosstalk between ATM and ATR, which has an adaptable component that responds to the DSB load and which has a connection to the cell cycle through Chk1 - and possibly other kinases. Notably, similar connections between resection and checkpoint and a defining role of Ku in the overall response has been reported for *Saccharomyces* cells suffering a single DSB and were found to affect recovery from the checkpoint^105^.

In aggregate, our observations demonstrate contributions of DNA-PKcs, ATM and ATR to the regulation of resection and G_2_-checkpoint that are consistent with crosstalk and possibly cross-regulation among them. We postulate therefore their functional integration in a regulatory module that detects DSBs, guides DSBs through the various stages of processing, and interphases DSB detection and processing with the diverse manifestations of DDR.

## Methods

### Cell culture and irradiation

Cells were grown in 10–20% fetal bovine serum (FBS)-supplemented McCoy’s 5A, MEM, D-MEM or RPMI cell culture media as outlined below, at 37 °C in a humidified atmosphere of 5% CO_2_ in air. A549, HCT116-DNA-PKcs^−/−^ and parental HCT116 cells were maintained in McCoy’s 5A medium; the ATM mutant cell lines, AT5BIVA and AT hTert (GM2052), as well as the conditionally ATR deficient, ATR GM847-ATRkd, the DNA ligase IV deficient, 180BRM, the XLF deficient, P2 hTert, cells and the normal human fibroblast 82-6 hTert cells were maintained in minimum essential medium (MEM). The DNA-PKcs deficient, M059J and their wild-type counterparts, M059K, cells were maintained in Dulbecco’s modified MEM (D-MEM). A human fibroblast cell line derived from a Seckel-Syndrome patient, F02-98, was grown in MEM, while a human lymphoblast cell line derived from a Seckel-Syndrome patient, DK0064, was grown in RPMI. For experiments designed to determine the mitotic index, cells were exposed to IR at 37 °C using a 320 kV X-ray machine with a 1.65 Al filter (GE Healthcare). The dose rate at 500 mm distance from the source was 2.7 Gy/min. For all remaining experiments, cells were exposed to IR at room temperature (RT).

### Treatment of cells with kinase inhibitors

Caffeine (Sigma-Aldrich) was dissolved in distilled water at 100 mM and was used at a final concentration of 4 mM. 2-morpholin-4-yl-6-thianthren-1-yl-pyran-4-one (KU55933, ATMi, Calbiochem) was dissolved in DMSO (Sigma-Aldrich) at 10 mM and was used at 10 μM final concentration. 7-hydroxystaurosporine (UCN-01, Chk1i, Calbiochem) was dissolved in DMSO at 100 μM and was used at 100 nM final concentration. The Chk2 Inhibitor (Chk2 Inhibitor-II/BML-277, Chk2i, Calbiochem) was dissolved in DMSO at 1 mM and used at a final concentration of 400 nM. 8-(4-Dibenzothienyl)-2-(4-morpholinyl)-4H-1-benzopyran-4-one (NU7441, DNA-PKi, Tocris Bioscience) was dissolved in DMSO at 10 mM and was used at a 10 μM final concentration. 3-Amino-6-[4-(methylsulfonyl)phenyl]-N-phenyl-2-pyrazinecarboxamide (ATRi, VE-821, Haoyuan Chemexpress) was dissolved in DMSO at 10 mM concentration and was used at a 5 μM final concentration, unless indicated otherwise. All inhibitors were added to the cells 1 h before irradiation and were maintained until collection for analysis. The IC_50_ values of these inhibitors for their targets and related PIKKs are as follow: ATRi (IC_50,ATR_ = 26 nM, IC_50,ATM_ = >8 μM, IC_50,DNA-PKcs_ = 4.4 μM), ATMi (IC_50, ATR_ = 100 μM, IC_50, ATM_ = 13 nM, IC_50, DNA-PKcs_ = 2.5 μM), DNA-PKcsi (IC_50, ATR_ = 100 μM, IC_50, ATM_ = 100 μM, IC_50, DNA-PKcs_ = 13 nM), Chk1i (IC_50_ = 12.5 nM), and Chk2i (IC_50, Chk2_ = 15 nM).

To induce the expression of ATR kinase-dead (ATRkd) protein that exerts a dominant-negative function on ATR, exponentially growing GM847-ATRkd cells were exposed for 48 h to Doxycycline Hyclate (DOX), (Sigma-Aldrich) at a concentration of 3 μg/ml. DOX was maintained in the cultures during the entire experiment.

### RNA interference

To suppress the activity of ATR, commercially available specific siRNA (QIAGEN) against ATR (Hs_ATR_11 FlexiTube siRNA), was utilized. The siRNAs were delivered to the cells by nucleofection, using the Nucleofector 2D device (Lonza Bioscience). The efficiency of the knock-down was assessed by quantitating protein levels using western blot analysis, 24 h after nucleofection.

### Flow cytometry analysis of mitotic index using H3-pS10 staining

Bivariate flow cytometry was employed to simultaneously measure DNA content by propidium iodide (PI) staining and mitotic cells by quantification of the phosphorylated Histone H3 at Serine 10 (H3-pS10). Briefly, 0.6–1.0 × 10^6^ cells were fixed in 70% ice-cold ethanol and were permeabilized for 15 min in ice-cold PBS supplemented with 0.25% Triton X-100. Cell pellets were incubated in 0.05% Tween-20, 1% BSA in PBS for 45 min at RT followed by incubation with an anti-H3-pS10 specific antibody (Abcam) for 2 h at RT. Cells were washed three times with PBS and incubated in AlexaFluor 488-conjugated goat-anti rabbit-IgG (Thermo Fisher Scientific). Finally, DNA was stained with PI for 30 min at 37 °C. All incubation steps were performed under gentle agitation. Analysis was carried out in a Gallios flow cytometer (Beckman Coulter) by measuring 2 × 10^4^ cells per sample; proper gating was applied to select H3-pS10 positive events that represent mitotic cells. The mitotic index (MI) was determined as the fraction of cells in mitosis and is shown normalized to the MI of non-irradiated controls. The actual MIs of the controls used for the normalization are given in the legends of the corresponding figures.

### Indirect immunofluorescence (IF) and image analysis

For immunofluorescence analysis, cells were grown on poly-L-lysine (Biochrom) coated coverslips. For analysis using BrdU detection, cells were pre-incubated for 24 h in media containing 10 μM BrdU. When required, S-phase cells were labeled with 10 μM of 5-ethynyl-2′-deoxyuridin (EdU) for 30 min. After irradiation, cells were washed three times with ice-cold PBS and fixed in PFA solution, (3% paraformaldehyde and 2% sucrose), for 15 min at RT. Subsequently, cells were washed three times with PBS and permeabilized in P-solution (100 mM Tris pH 7.4, 50 mM EDTA, 0.5% Triton X-100) for 10 min at RT. After washing three times with PBS, samples were blocked in PBG solution (0.2% skin fish gelatin, 0.5% BSA fraction V, in PBS) overnight at 4 °C. Primary antibodies, pATM-S1981 (Cell Signaling Technology, 4526), pChk2-T68 (BIOZOL, BZL08950), Cyclin B1 (Santa Cruz Biotechnology, H-433), 53BP1 (Santa Cruz Biotechnology, H-300) and RPA70 (αSSB70B, mouse hybridoma cell line kindly provided by Dr. G. Hurwitz)^118^ were diluted (1:300) in PBG solution, while the anti-BrdU antibody (BD) was used at a dilution of 1:100. The cover slips were incubated at RT for 2 h and washed three times with PBS-T (0.05% Tween-20 in PBS). Alexa Fluor-conjugated secondary antibodies, anti-mouse IgG Alexa Fluor 488 and anti-rabbit IgG Alexa Fluor 568 (Thermo Fisher Scientific, A11001; A11011), were applied at 1:400 dilution for 1 h at RT. When necessary, the EdU signal was developed using an EdU staining kit (Thermo Fisher Scientific) according to the manufacturer’s instructions. Finally, cells were counterstained with 100 ng/ml DAPI (Thermo Fisher Scientific) at RT for 5 min and coverslips were mounted in PromoFluor antifade reagent (PromoCell). Scanning was carried out on a Leica TCS-SP5 confocal microscope (Leica Microsystems). For each slide, 5 fields were scanned (about 200 nuclei) and the Z-stacks were processed using Imaris image analysis software (Bitplane). Immunofluorescence analysis of EdU labeled cells was carried out using a high-content imaging and image analysis platform (Metasystems). In total ~1600 cells were analyzed to obtain 100–150 EdU-negative, G_2_-phase cells for the quantification of parameters of interest.

### Flow cytometry analysis of DNA end-resection by Rpa70 or BrdU signal quantification

For DNA end-resection analysis using Rpa70 detection, exponentially growing cells were pulse-labeled for 30 min with 10 μM EdU. For DNA end resection analysis using BrdU detection, cells were pre-incubated for 24 h in media containing 10 μM BrdU, labeled with EdU and processed as described next. After EdU incubation the growth medium was removed and cells were rinsed once with pre-warmed PBS. Fresh medium containing or not PIKK inhibitors was supplied for 1 h and cells were exposed to X-rays. At different times thereafter, cells were collected by trypsinization and unbound RPA was extracted by incubating the cell pellets for 5 min in ice-cold PBS containing 0.2% Triton™ X-100. Cells were spun-down for 5 min and pellets were fixed for 15 min with 3% PFA plus 2% sucrose dissolved in PBS. Cells were blocked with PBG blocking buffer overnight at 4 °C and incubated for 1.5 h with a monoclonal antibody raised against Rpa70 (αSSB70B, mouse hybridoma cell line kindly provided by Dr. J. Hurwitz^118^), or an anti-BrdU monoclonal antibody (BD, 347580). Cells were washed twice with PBS and incubated for 1.5 h with a secondary antibody conjugated with AlexaFluor 488 (Thermo Fisher Scientific, A11001). Subsequently, EdU signal was developed using an EdU staining kit (Thermo Fisher Scientific) according to the manufacturer’s instructions. Three-parameter analysis was carried out with a Gallios flow cytometer (Beckman Coulter) and quantitated using the appropriate software as outlined in Fig. 5A (Kaluza 1.3, Beckman Coulter).

### Polyacrylamide gel electrophoresis (SDS-PAGE) and western blotting

Cells were collected and washed twice in ice-cold PBS. Approximately 5 × 10^6^ cells were lysed for 30 min in 0.2–0.5 ml of ice-cold RIPA buffer (Thermo Fisher Scientific) supplemented with Halt phosphatase and protease inhibitor cocktails (Thermo Fisher Scientific), as recommended by the manufacturer. Lysates were spun-down for 15 min at 12,000 × g, 4 °C and protein concentration was determined in the supernatants using the Bradford assay. Standard protocols for SDS-PAGE and immunoblotting were employed. Unless otherwise indicated, 40 μg RIPA whole cell extract was loaded in each lane. The primary antibodies were: anti-Ku80 (Santa Cruz Biotechnology, sc-9034), anti-Ku70 (GeneTex, GTX77607 and GTX23114), anti-ATR (Santa Cruz Biotechnology, sc-28901), anti-ATRIP (mouse hybridoma cell line kindly provided by Dr. Halazonetis), anti-pATM-S1981 (Cell Signaling Technology, 4526), anti-pChk2-T68 (BIOZOL, BZL08950), anti-pChk2-S256 (Santa Cruz Biotechnology, sc-101658), anti-Chk2 (Santa Cruz Biotechnology, sc-9064), anti-p53-pS15 (GeneTex, GTX21431), anti-GAPDH (MERCK, MAB374), anti-Rpa32 (mouse hybridoma cell line kindly provided by Dr. G. Hurwitz)^118^, anti-pKAP1-p824 (Bethyl Laboratories, A300–767A) and anti-DNA-PKcs (MERCK, PC127) were used at 1:500 to 1:10000 dilution. The secondary antibodies were anti-mouse IgG conjugated with IRDye680 or anti-rabbit-IgG conjugated with IRDye800 (LI-COR Biosciences, 92668020 and 92632211) at 1:15,000 dilution. Immunoblots were visualized by scanning membranes in an Odyssey infrared scanner (LI-COR Biosciences). When applicable the raw pseudo colored western blot images were converted to grayscale by Odyssey imaging software. Additionally, western blot images were processed by using the brightness and contrast functions of the Odyssey software; raw data can be found in Fig. S6.

## Supplementary information

Supplementary information
