# Supplementary material for: Radiation-dose-dependent functional synergisms between ATM, ATR and DNA-PKcs in checkpoint control and resection in G2-phase
Source: Sci Rep. 2019 Jun 4;9:8255. doi: 10.1038/s41598-019-44771-6 (PMC6547644; doi:10.1038/s41598-019-44771-6)
Supplement: Supplementary file 1 — Supplementary information [file 41598_2019_44771_MOESM1_ESM.pdf]

Supplementary Information

Radiation-dose-dependent functional synergisms between ATM, ATR and DNA-PKcs in checkpoint control and resection in G2-phase

Emil Mladenov, Xiaoxiang Fan, Rositsa Dueva, Aashish Soni and George Iliakis

Supplementary Figure Legends

Fig. S1: (A) Normalized MI as a function of time in untreated and ATR, ATM and DNAPK inhibitor treated A549 cells exposed to 4 Gy. Other details as in Figure 1A. **(B)** As in A, for GM847-ATRkd cells analyzed in the absence of doxycycline (-DOX) after exposure to 4 Gy in the presence or absence of ATMi or ATRi. **(C)** As in A, for untreated AT hTert cells exposed to 2 Gy and incubated with ATMi or ATRi. **(D)** As in A, for A549 cells exposed to 1 Gy in the presence of absence of Chk1i, or Chk2i. Data represent the mean and standard deviation calculated from three independent experiments.

Fig. S2: (A) Western blot analysis of Chk2-pS516 and Chk2-pT68 in untreated A549 cells or cells treated with ATMi or Chk2i, 1 h after exposure to 4 Gy. GAPDH, Ku70 and non-phosphorylated Chk2 serve as loading controls. **(B)** IF analysis of ATM-pS1981 and Chk2-pT68 foci in A549 cells, 1 h after exposure to 4 Gy and treated with ATMi, ATRi or Chk1i as indicated. **(C)** Western blot analysis of ATM-pS1981, Chk2-pT68 and p53-pS15 in A549 cells treated with ATRi or ATMi and exposed to 4 Gy. GAPDH and non-phosphorylated Chk2 serve as loading controls. **(D)** Normalized MI as a function of time in AT hTert cells treated with the indicated PIKK inhibitors and exposed to 10 Gy.

Fig. S3: (A) Histograms of Rpa70 signal intensity as a function of time in 82-6 hTert cells exposed to 10 Gy in G_2-phase and incubated in the presence of different concentrations of ATRi for 1 or 3 h. **(B)** Histograms of Rpa70 signal intensity as a function of time in A549 cells exposed to 10 Gy in G_2-phase and incubated in the presence or absence of ATRi. **(C)** As in B, for AT hTert cells.

Fig. S4: (A-C) Normalized MI as a function of time in DNA-PKcs deficient, HCT116-DNAPKcs$^{-/-}$, M059K and wild-type HCT116 cells, exposed to 2 Gy and treated with the indicated PIKK inhibitors**. (D)** Western blot analysis of DNA-PKcs, ATM, CtIP, Mre11 and Ku70 in M059K and M059J cells. **(E)** Western blot analysis of p53-pS15, KAP1-pS824 and Chk2-pT68 in M059K and M059J cells exposed to 0 or 4 Gy and treated with the indicated PIKK inhibitors. GAPDH, ATRIP, DNA-PKcs, Ku70 and Ku80, as well as non-phosphorylated Chk2 are used as loading controls. **(F)** IF analysis of ATM activation in M059K and M059J cells treated with the indicated PIKK inhibitors and exposed to 2 Gy. ATM-pS1981 foci analysis is carried out specifically in late-S/G_2 cells identified by positive Cyclin B1 staining (red). **(G)** As in A, for HCT116-DNAPKcs$^{-/-}$ cells, treated with indicated PIKK and exposed to 10 Gy.

Fig. S5: (A) Histograms of Rpa70 signal intensity at 6 h in M059K cells exposed to 10 Gy in G_2-phase and incubated in the presence or absence of ATRi or Chk1i. **(B)** Histograms of Rpa70 signal intensity at 6 h in HCT116-DNAPKcs$^{-/-}$ cells exposed to 10 Gy in G_2-phase and incubated in the presence or absence of ATMi, ATRi and ATMi + ATRi. **(C)** Histograms of Rpa70 signal intensity as a function of time in 82-6 hTert cells exposed to 10 Gy in G_2-phase and incubated in the absence of inhibitors, or with a cocktail of inhibitors including ATMi, ATRi and DNA-PKcsi. **(D)** Histograms of Rpa70 signal intensity as a function of time in 82-6 hTert, XLF deficient, P2 hTert, or Lig4 deficient, 180BRMi, cells after exposure to 10 Gy in G_2-phase and incubated in the presence of ATRi.

Fig. S6: (A) Raw images of western blot analyses included in Fig. 1B and Fig. 1D **(B)** Raw images of western blot analyses included in Fig. 2C **(C)** Raw images of western blot analyses included in Fig. 2D **(D)** Raw images of western blot analyses included in Fig. 2E **(E)** Raw images of western blot analyses included in Fig. S2A **(F)** Raw images of western blot analyses included in Fig. S2C **(G)** Raw images of western blot analyses included in Fig. S4D **(H)** Raw images of the western blot analyses included in Fig. S4E

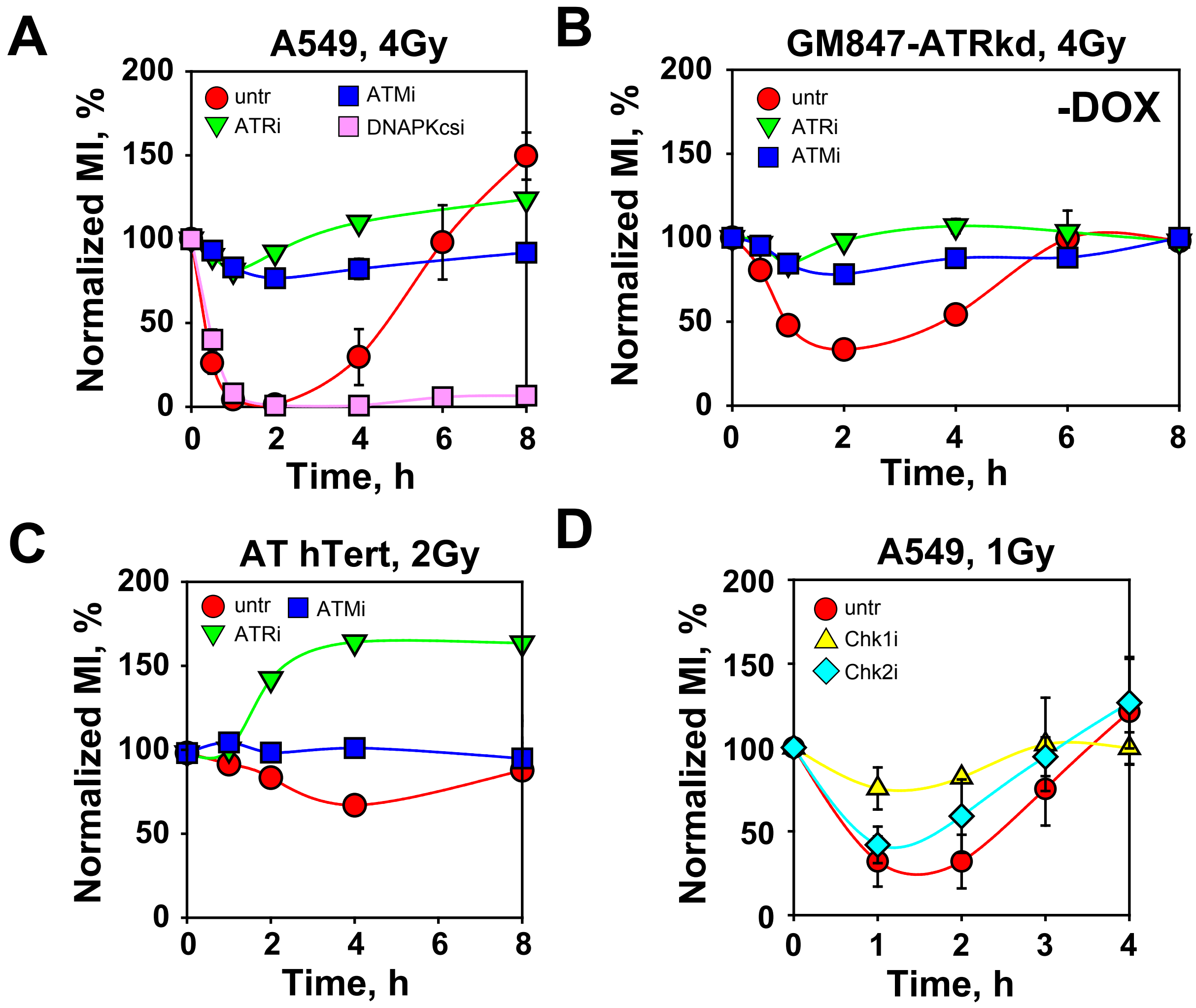
A
A549, 4Gy
untr
ATRi
ATMi
DNAPKcsi
Normalized MI, %
Time, h
B
GM847-ATRkd, 4Gy
-DOX
untr
ATRi
ATMi
Normalized MI, %
Time, h
C
AT hTert, 2Gy
untr
ATRi
ATMi
Normalized MI, %
Time, h
D
A549, 1Gy
untr
Chk1i
Chk2i
Normalized MI, %
Time, h

Fig. S1

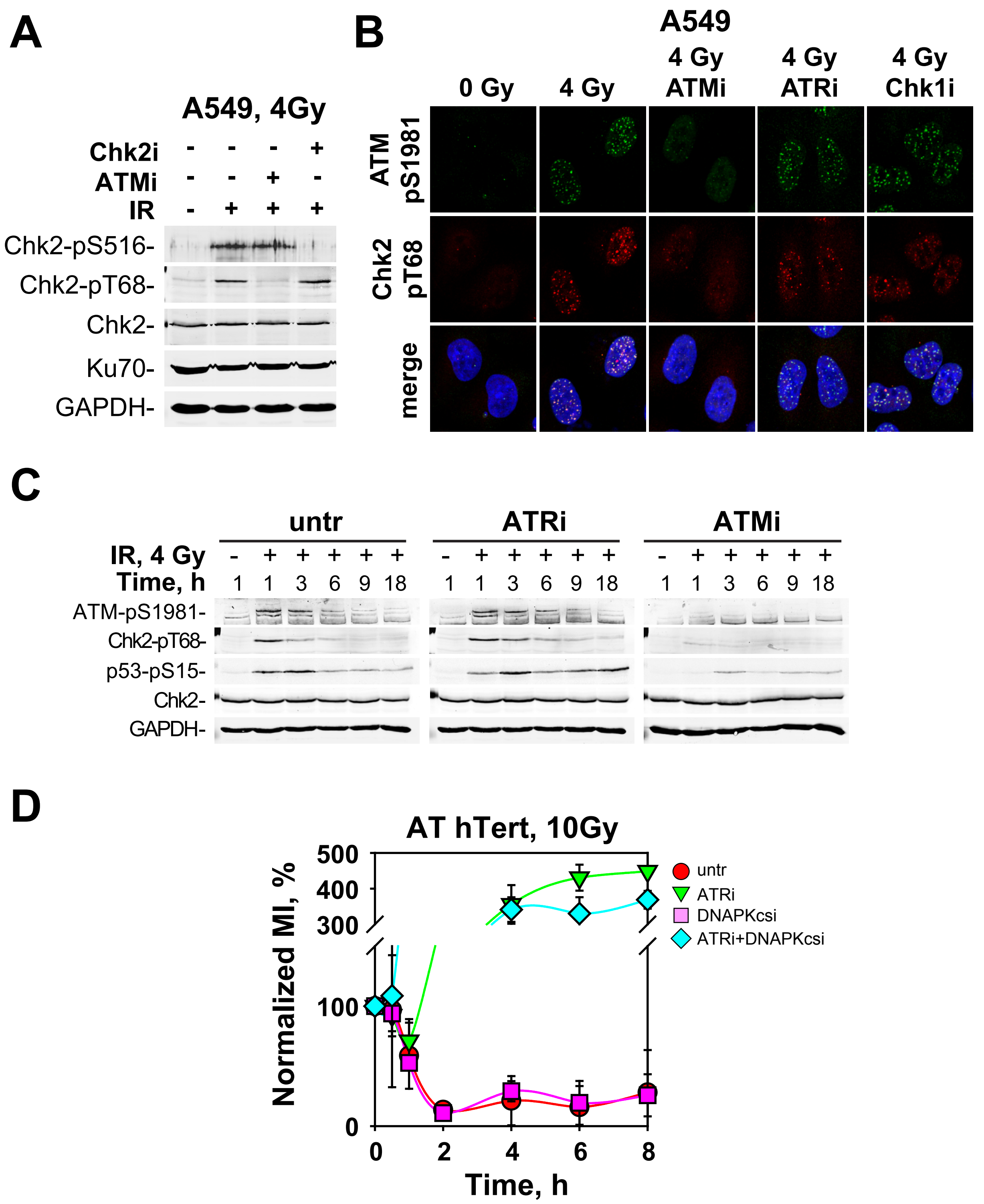

A
A549, 4Gy
Chk2i - - - +
ATMi - - + -
IR - + + +
Chk2-pS516-
Chk2-pT68-
Chk2-
Ku70-
GAPDH-
B
A549
0 Gy
4 Gy
4 Gy ATMi
4 Gy ATRi
4 Gy Chk1i
ATM pS1981
Chk2 pT68
merge
C
untr
ATRi
ATMi
IR, 4 Gy - + + + + +
Time, h 1 1 3 6 9 18
ATM-pS1981-
Chk2-pT68-
p53-pS15-
Chk2-
GAPDH-
D
AT hTert, 10Gy
Normalized MI, %
Time, h
untr
ATRi
DNAPKcsi
ATRi+DNAPKcsi

Fig. S2

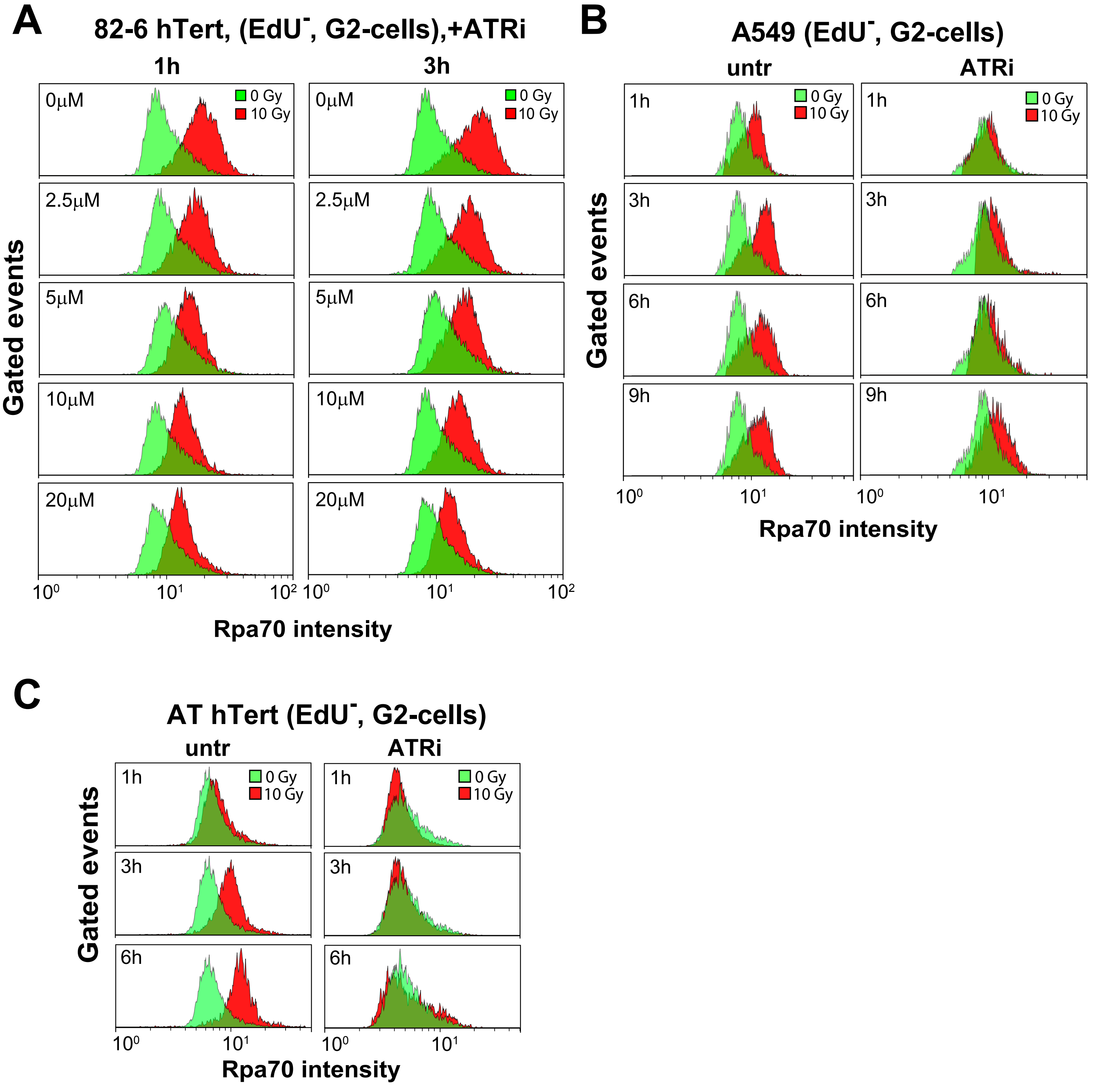
A
82-6 hTert, (EdU-, G2-cells),+ATRi
1h
3h
0μM
2.5μM
5μM
10μM
20μM
0 Gy
10 Gy
Gated events
Rpa70 intensity
B
A549 (EdU-, G2-cells)
untr
ATRi
1h
3h
6h
9h
0 Gy
10 Gy
Gated events
Rpa70 intensity
C
AT hTert (EdU-, G2-cells)
untr
ATRi
1h
3h
6h
0 Gy
10 Gy
Gated events
Rpa70 intensity

Fig. S3

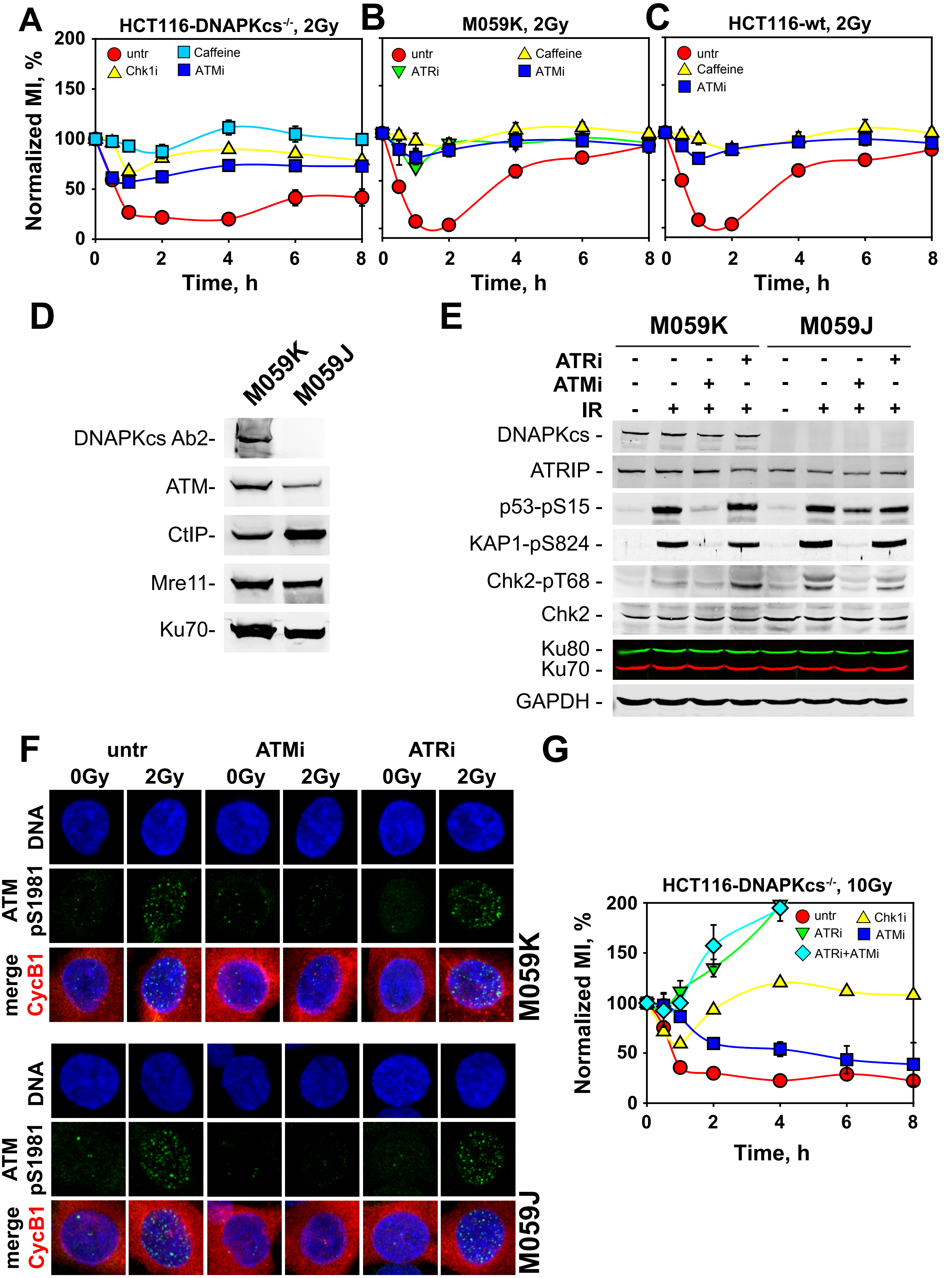
A
HCT116-DNAPKcs-/-, 2Gy
untr
Caffeine
Chk1i
ATMi
Normalized MI, %
Time, h
B
M059K, 2Gy
untr
Caffeine
ATRi
ATMi
C
HCT116-wt, 2Gy
untr
Caffeine
ATMi
D
M059K
M059J
DNAPKcs Ab2-
ATM-
CtIP-
Mre11-
Ku70-
E
M059K
M059J
ATRi
ATMi
IR
DNAPKcs -
ATRIP -
p53-pS15 -
KAP1-pS824 -
Chk2-pT68 -
Chk2 -
Ku80 -
Ku70 -
GAPDH -
F
untr
ATMi
ATRi
0Gy
2Gy
DNA
ATM pS1981
merge CycB1
M059K
M059J
G
HCT116-DNAPKcs-/-, 10Gy
untr
Chk1i
ATRi
ATMi
ATRi+ATMi
Normalized MI, %
Time, h

Fig. S4

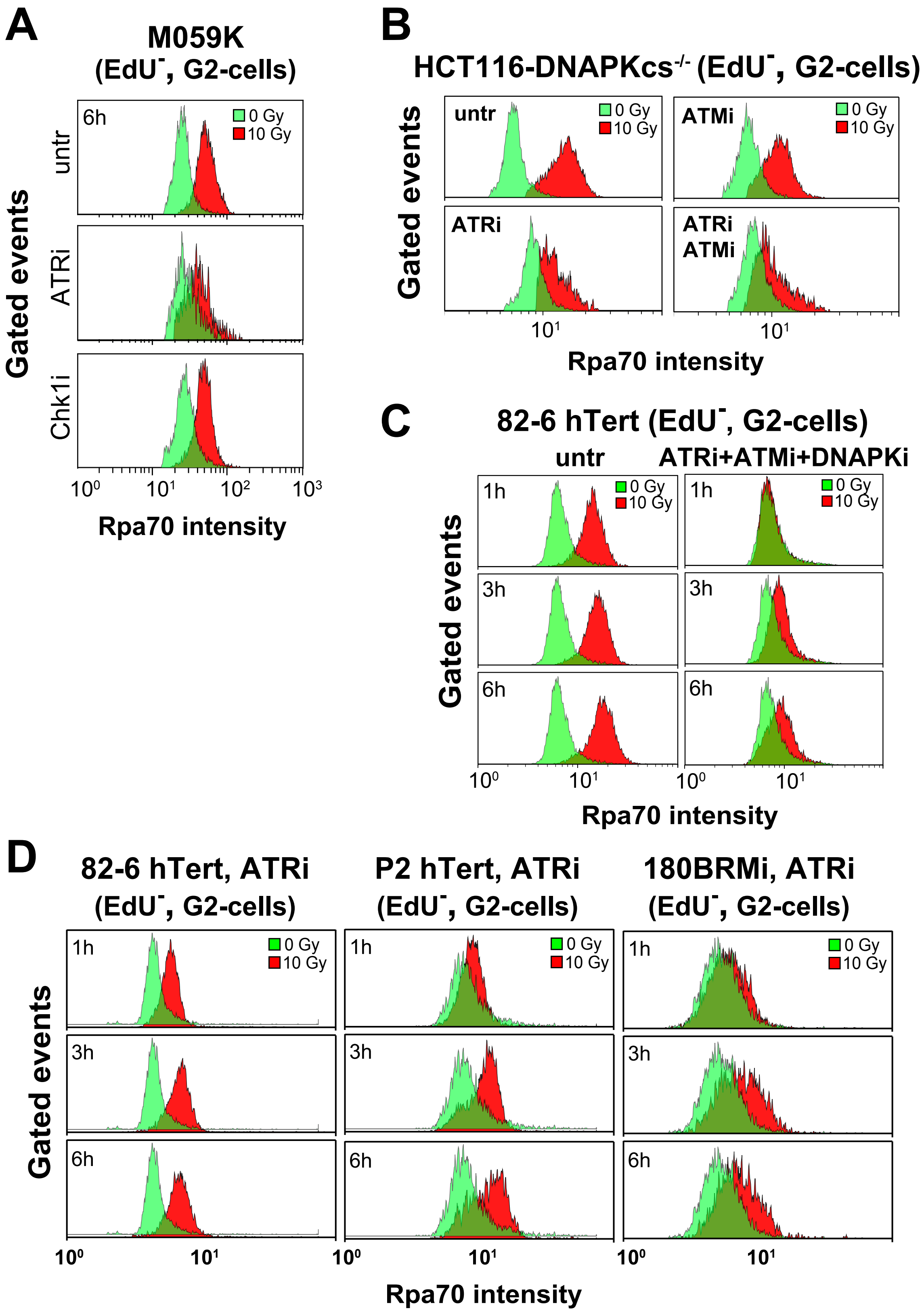

A
M059K
(EdU-, G2-cells)
Gated events
untr
ATRi
Chk1i
6h
0 Gy
10 Gy
10⁰ 10¹ 10² 10³
Rpa70 intensity
B
HCT116-DNAPKcs-/- (EdU-, G2-cells)
Gated events
untr
ATMi
ATRi
ATRi ATMi
0 Gy
10 Gy
10¹
Rpa70 intensity
C
82-6 hTert (EdU-, G2-cells)
untr
ATRi+ATMi+DNAPKi
Gated events
1h
3h
6h
0 Gy
10 Gy
10⁰ 10¹
Rpa70 intensity
D
82-6 hTert, ATRi (EdU-, G2-cells)
P2 hTert, ATRi (EdU-, G2-cells)
180BRMi, ATRi (EdU-, G2-cells)
Gated events
1h
3h
6h
0 Gy
10 Gy
10⁰ 10¹
Rpa70 intensity

Fig. S5

GM847-ATRkd

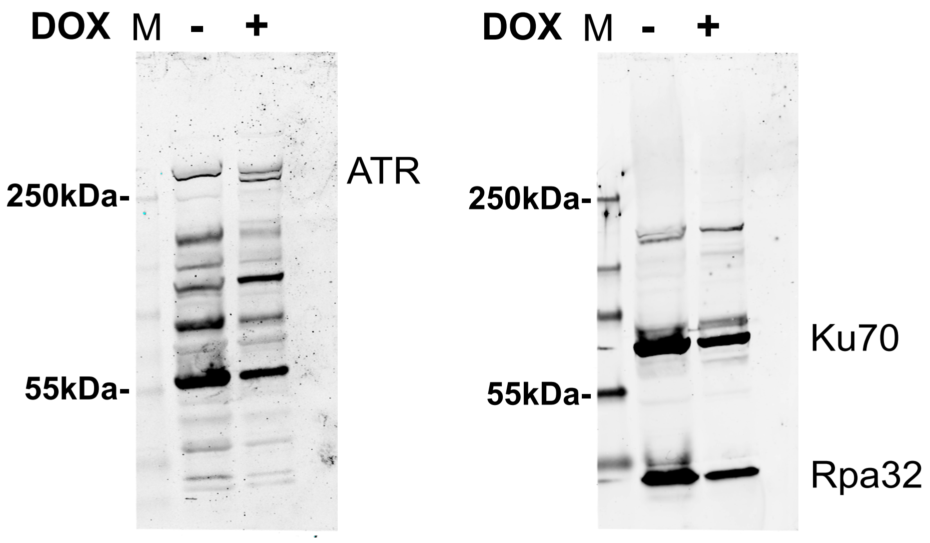

Fig. 1B

82-6 hTert

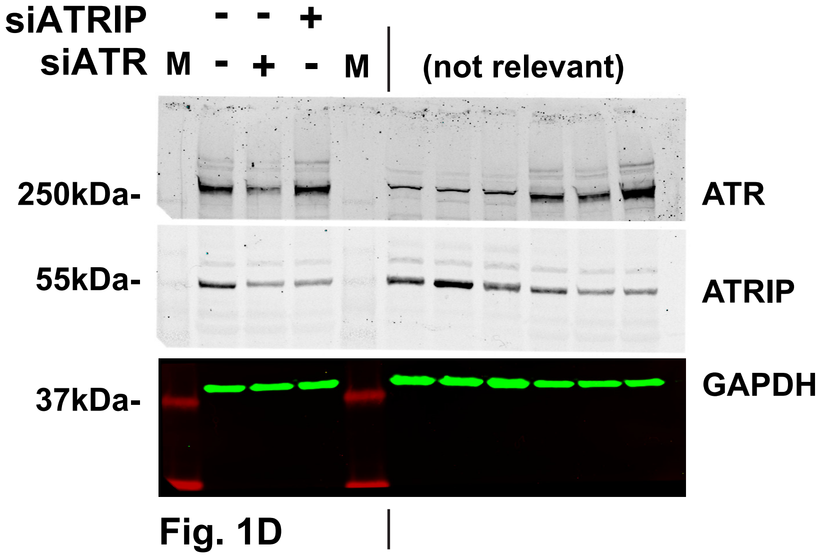

Fig. 1D

Fig. S6A

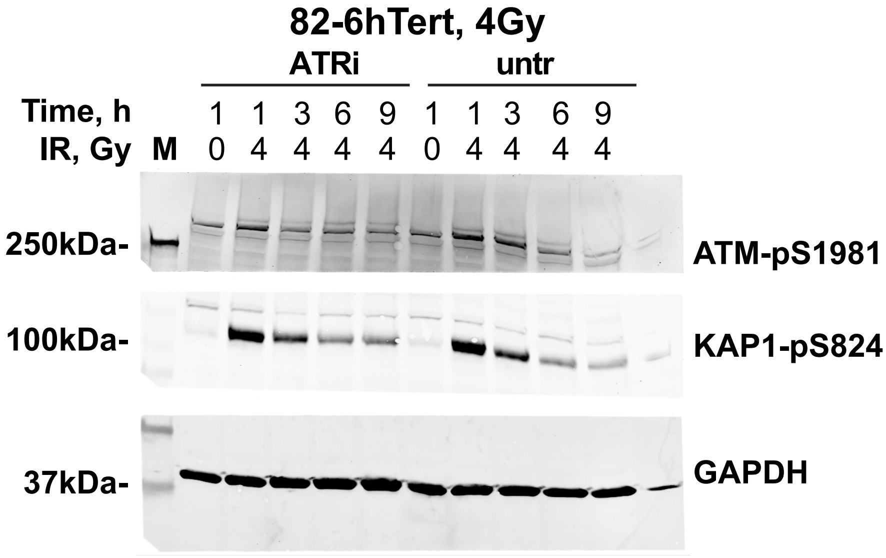

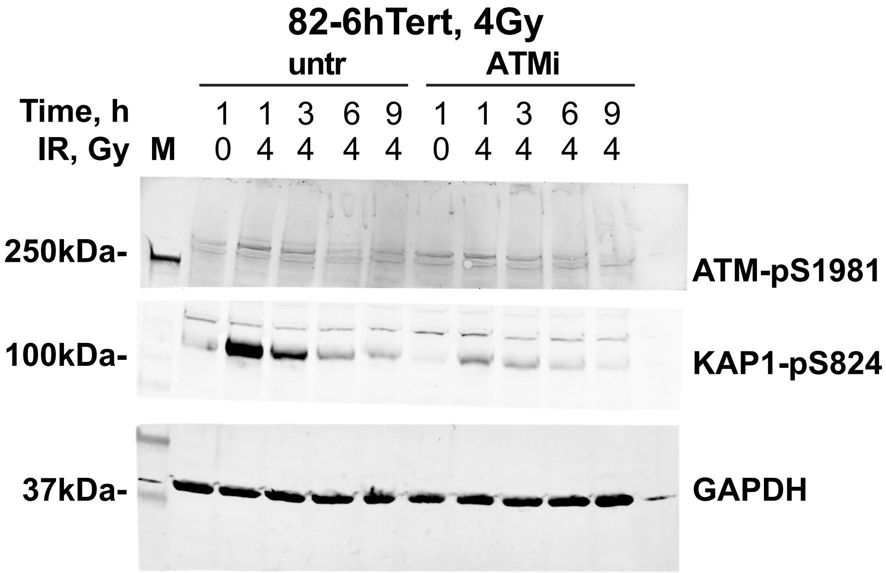

Fig. 2C

Fig. S6B

82-6hTert, 4Gy

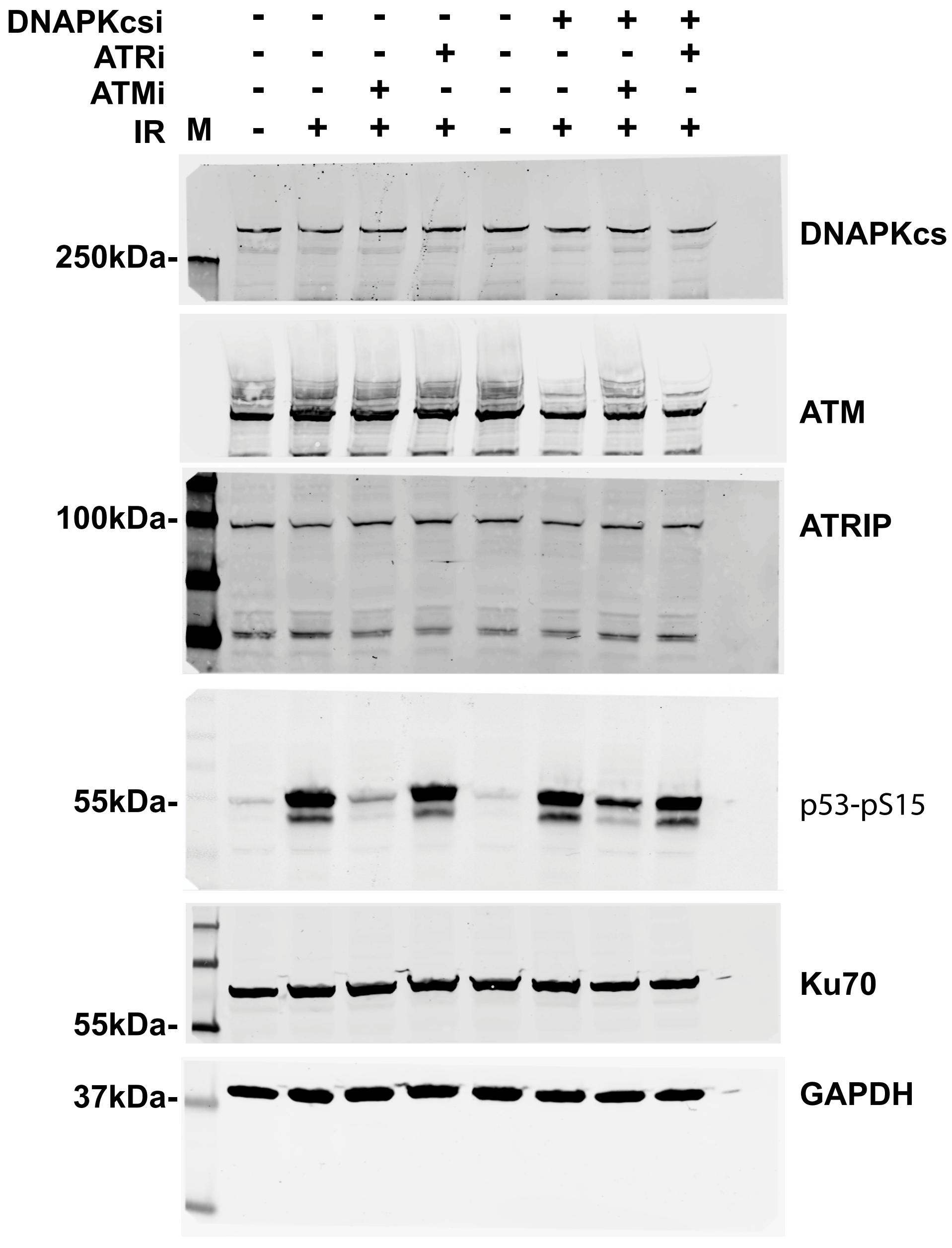

Fig. 2D

Fig. S6C

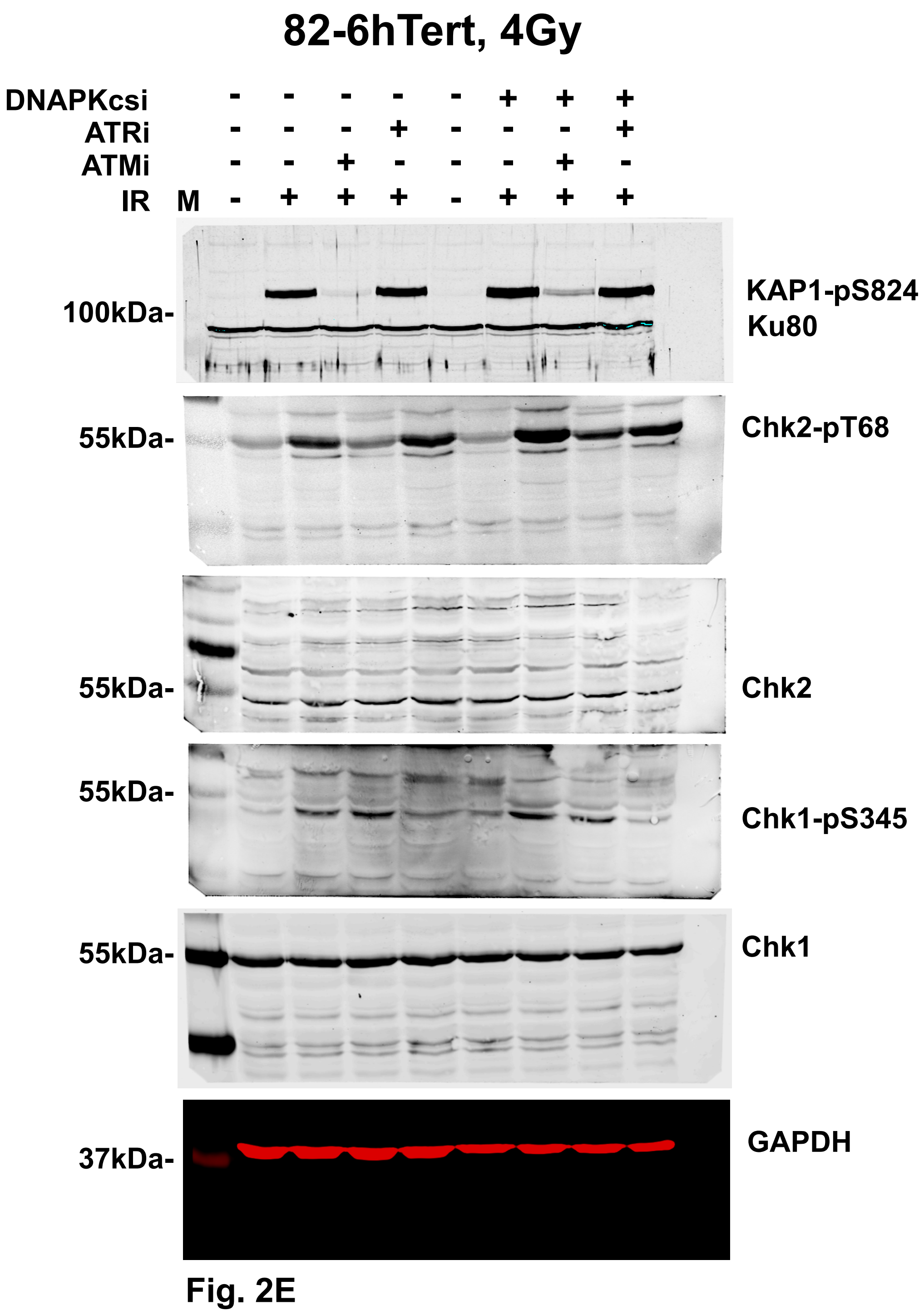
82-6hTert, 4Gy
DNAPKcsi - - - - - + + +
ATRi - - - + - - - +
ATMi - - + - - - + -
IR M - + + + - + + +
100kDa-
KAP1-pS824
Ku80
55kDa-
Chk2-pT68
55kDa-
Chk2
55kDa-
Chk1-pS345
55kDa-
Chk1
37kDa-
GAPDH
Fig. 2E

Fig. S6D

A549, 4Gy

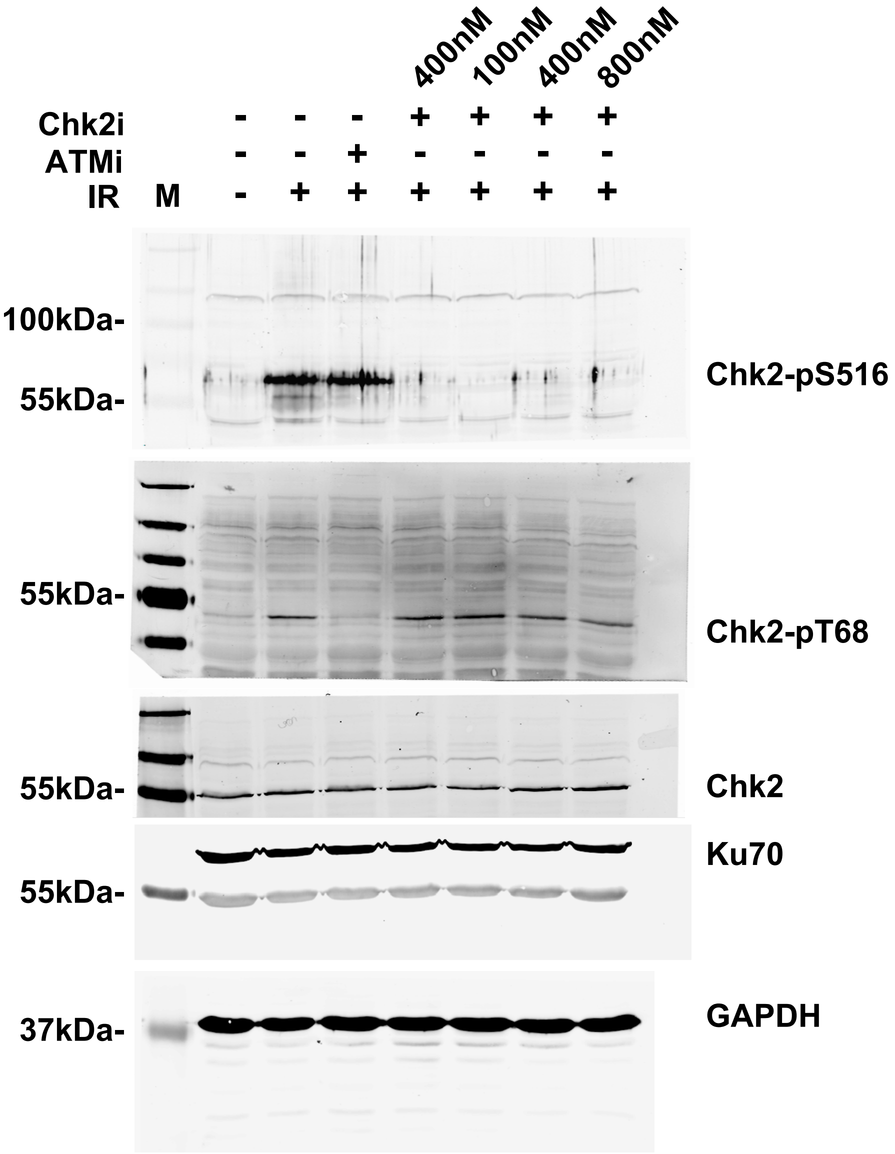

Fig. S2A

Fig. S6E

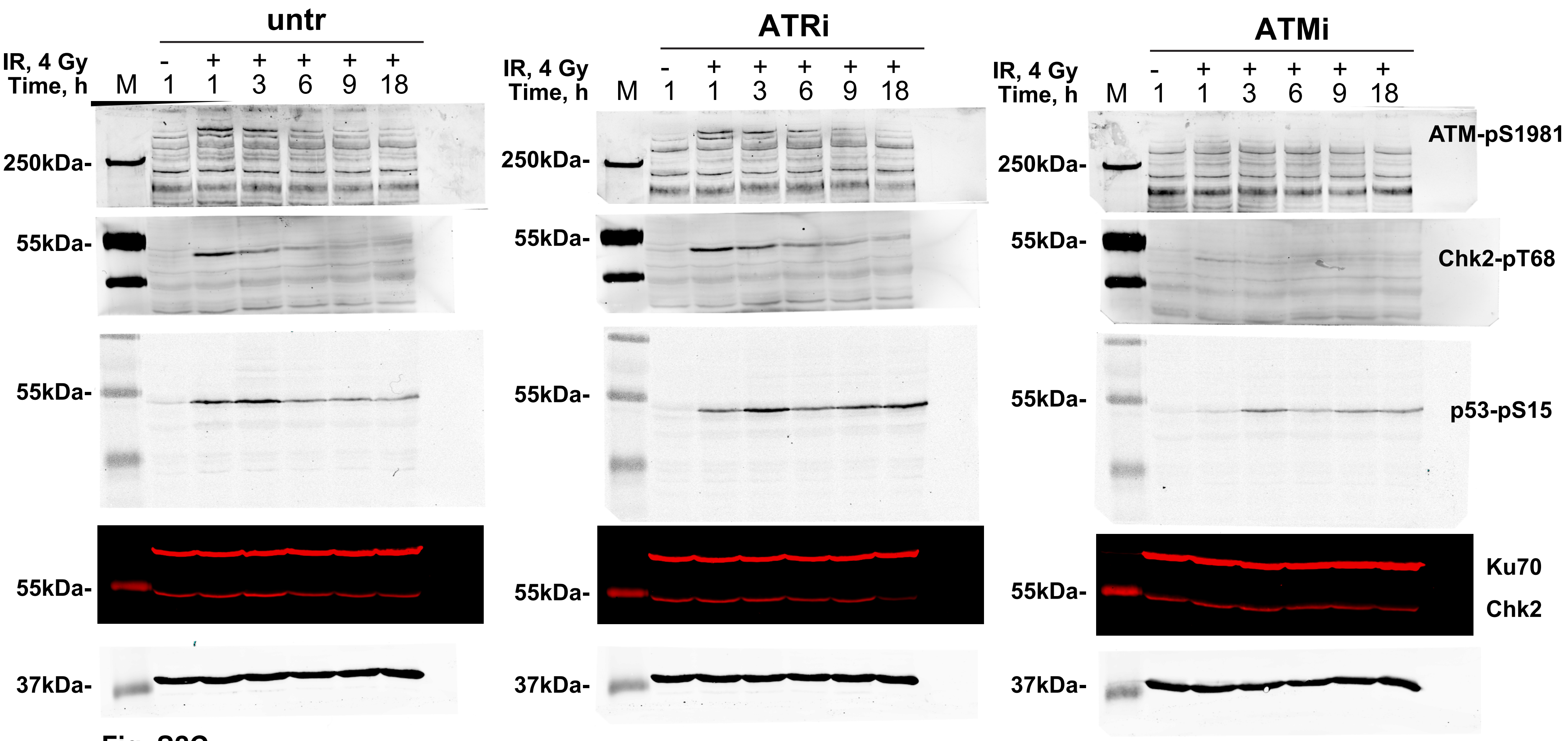

Fig. S2C

Fig. S6F

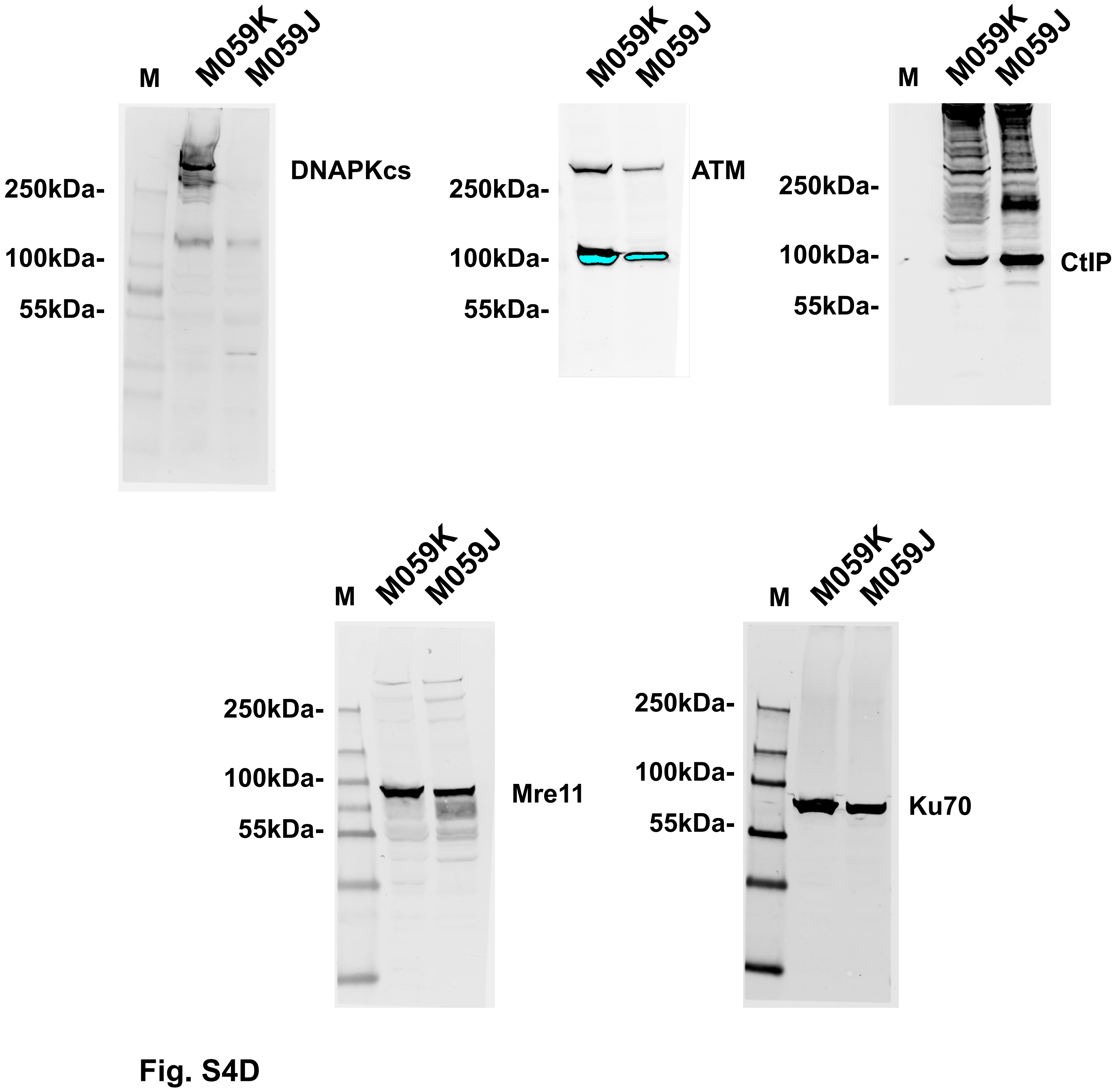

Fig. S4D

Fig. S6G

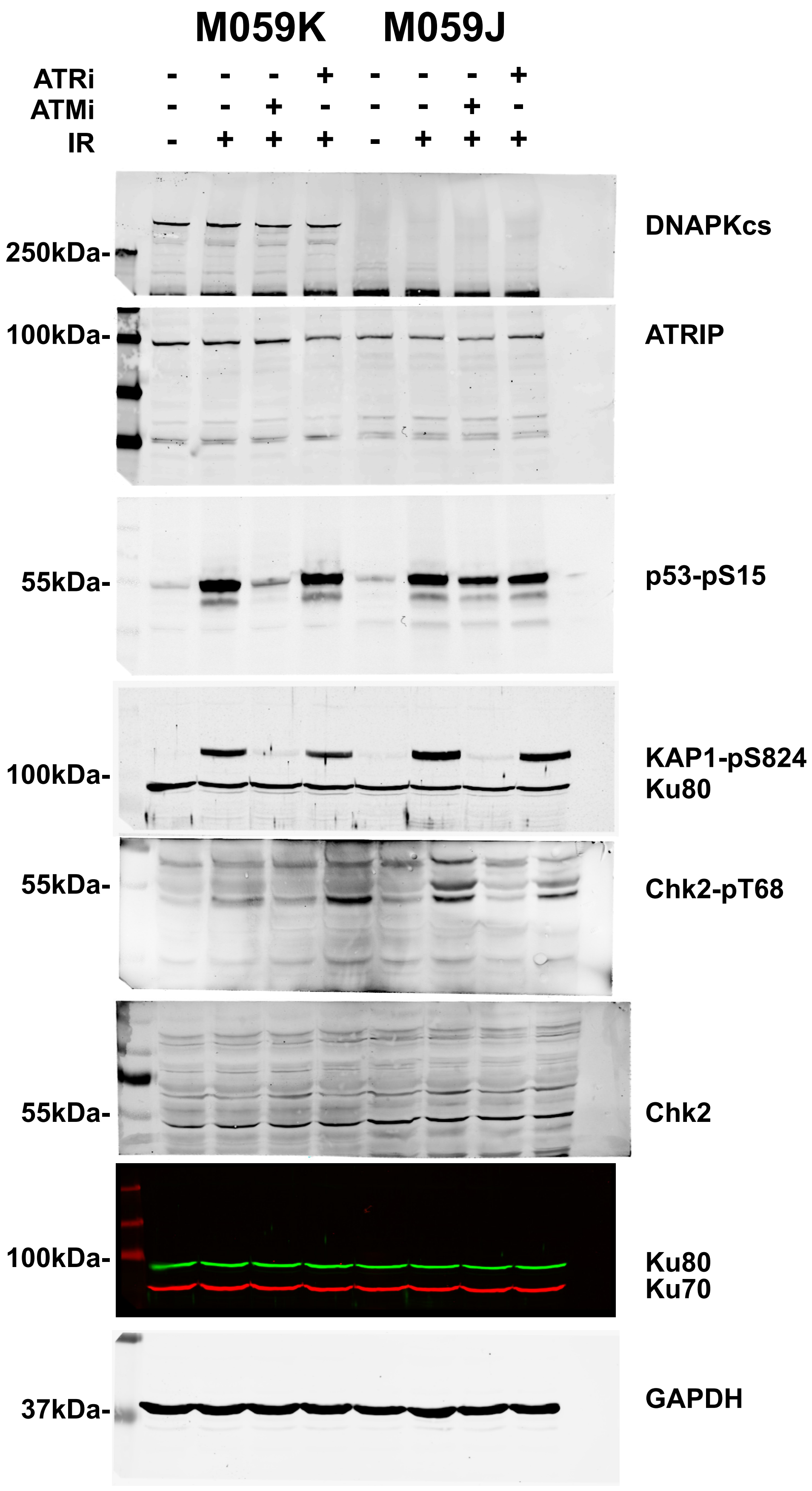

Fig. S4E

Fig. S6H
